# Fear in the Theater of the Mind: Differential Fear Conditioning With Imagined Stimuli

**DOI:** 10.1177/09567976221086513

**Published:** 2022-07-27

**Authors:** Lauryn Burleigh, Xinrui Jiang, Steven G. Greening

**Affiliations:** 1 Department of Psychology, Cognitive and Brain Sciences, Louisiana State University; 2 Department of Psychology, Brain and Cognitive Sciences, University of Manitoba

**Keywords:** fear conditioning, mental imagery, fear, learning, emotion, open data, open materials

## Abstract

Many symptoms of anxiety and posttraumatic stress disorder are elicited by fearful mental imagery. Yet little is known about how visual imagery of conditioned stimuli (CSs) affects the acquisition of differential fear conditioning. Across three experiments with younger human adults (Experiment 1: *n* = 33, Experiment 2: *n* = 27, Experiment 3: *n* = 26), we observed that participants acquired differential fear conditioning to both viewed and imagined percepts serving as the CSs, as measured via self-reported fear and skin conductance responses. Additionally, this differential conditioning generalized across CS-percept modalities such that differential conditioning acquired in response to visual percepts generalized to the corresponding imagined percepts and vice versa. This is novel evidence that perceived and imagined stimuli engage learning processes in very similar ways and is consistent with the theory that mental imagery is depictive and recruits neural resources shared with visual perception. Our findings also provide new insight into the mechanisms of anxiety and related disorders.

For more than 100 years, differential fear conditioning has been an influential paradigm for understanding fear (Dvir et al., 2019; Mechias et al., 2010; Watson & Rayner, 2000). It involves two neutral conditioned stimuli (CSs), one of which (CS+) is paired with an aversive unconditioned stimulus (US; e.g., mild electrical stimulation) whereas the other (CS–) is never paired with the US. Differential conditioning is acquired when the CS+ produces a larger conditioned response than the CS− as measured, for example, via the skin conductance response (SCR) or self-reported fear. More recently, interest has grown in how higher-order processes such as working memory (Carter et al., 1998), concepts (Boyle et al., 2016; Grégoire & Greening, 2020), and mental imagery (Mertens et al., 2020; Mueller et al., 2019) contribute to fear conditioning.

Mental imagery plays an important role in emotions such as fear. Anxiety and posttraumatic stress disorder are associated with symptoms elicited by mental imagery, such as intrusive memories and flashbacks (Arntz et al., 2007). Converging evidence from research on mental imagery and emotion indicates that imagery may be depictive in addition to propositional (Ji et al., 2016; Lang, 1979; Pearson et al., 2015). According to the *depictive theory*, mental images activate early perceptual representations without external input (Pearson et al., 2015). Whereas a *visual percept* is a mental representation elicited by an external visual stimulus, an *imagined percept* is a representation elicited internally that operates as a form of the corresponding visual percept and is associated with subjective vividness, as if one is “seeing with the mind’s eye.” The depictive theory predicts that differential fear conditioning to visual percepts generalizes to the corresponding imagined percepts, despite the latter never being paired with the US, and vice versa.

Holzman and Levis (1991) appear to have provided the first evidence that conditioning to visual objects can generalize to imagery of the same objects; however, their generalization effect was observed during an extinction phase in which only data from CS+ trials were analyzed. Several recent studies provide additional evidence that differential fear conditioning acquired to visual percepts generalizes to imagined percepts of the same CS. For example, following the acquisition of differential fear conditioning to visual percept, mental imagery of the CS can contribute to both fear extinction (Agren et al., 2017; Jiang & Greening, 2021; Reddan et al., 2018) and reconsolidation (Grégoire & Greening, 2019), as measured primarily via SCR. Consistent with the depictive theory, findings reported by Greening et al. (2022) show both that imagery elicited significant differential reactivity, as measured with SCRs, self-reported fear, and anterior insula activity, and that imagery of the CSs was associated with significant multivoxel pattern classification in early visual cortices.

If mental imagery of the CSs is depictive, one would also predict that differential conditioning can be acquired to imagined CSs and that such conditioning should generalize to the corresponding visual percepts. After such an imagery-acquisition phase, Holzman and Levis (1991) observed that participants had significantly greater SCRs to the CS+ when imagining but not when viewing the CS+ during early extinction. This suggests that imagery acquisition is possible but that it might not generalize to the corresponding visual stimulus. However, Holzman and Levis analyzed only CS+ trials during the early extinction phase and failed to find evidence of successfully conditioned fear to imagined stimuli during acquisition. Regarding generalization, Lewis et al. (2013) more recently used evaluative conditioning to demonstrate that after imagined CSs were paired with negative and positive images, participants who viewed those CSs produced more significant reaction time priming effects when responding to congruent emotional images than to incongruent emotional images (Lewis et al., 2013). These findings provide some evidence that differential conditioning to imagined percepts and the generalization of such conditioning to corresponding visual percepts are possible.

Our primary goal in the current study was to determine whether mental imagery affects the processes of differential fear conditioning in a manner consistent with depictive theory. Across three experiments combining fear conditioning with mental imagery and visual perception, we addressed this goal by testing the following three hypotheses: (a) Differential fear conditioning acquired to visual percepts generalizes to matching imagined percepts, (b) differential fear conditioning can be established to imagined percepts, and (c) differential fear conditioning acquired to imagined percepts generalizes to matching visual percepts. All three experiments used within-subjects designs and measured two primary dependent variables: self-reported fear and SCR.

**Statement of Relevance**

From fears of monsters in the closet to the internal replay of traumatic events of our past, mental imagery plays an important role in the acquisition and generalization of fear responses. Visual mental imagery also engages brain areas that support visual perception. Largely unknown is how mental imagery is affected by emotional learning, in which fear responses are acquired to otherwise neutral imagined objects. We found that fear responses can be acquired when one is both viewing and imagining distinct objects, as measured with self-reports and sweat responses. Additionally, after the acquisition of fear for either visual or imagined objects, the fear response generalized to the corresponding imagined or visual objects, respectively. Our findings imply that visual perception and mental imagery engage similar neural circuits during fear learning and generalization. They also provide novel insight into how mental imagery could potentially influence the acquisition and symptom expression of anxiety-related disorders.

In Experiment 1, participants completed two differential-fear-acquisition phases in which they were cued auditorily to view and imagine both a CS+ and a CS−. In the *visual-acquisition phase*, 50% of trials on which participants were instructed to view the CS+ included mild electrical stimulation (the US) that ended at the same time as the Gabor patch disappeared, whereas trials on which they were instructed to imagine the CS+ were never paired with the US. In the imagery-acquisition phase, 50% of trials on which they were instructed to imagine the CS+ included the US, whereas trials on which they were instructed to view the CS+ were never paired with the US. Experiments 2 and 3 replicated the visual-acquisition and imagery-acquisition phases, respectively, ruling out the auditory prompt in Experiment 1 as a potential confound. Whereas visual trials in the follow-up experiments began with the presentation of a CS without auditory cues, imagery trials retained the auditory cue to indicate which CS participants should imagine. In Experiments 2 and 3, the auditory cue could be ruled out as a potential confound if differential fear conditioning acquired for one percept modality generalized to the other percept modality that was not paired with the US (i.e., visual acquisition generalized to imagined stimuli in Experiment 2, and imagery acquisition generalized to visual stimuli in Experiment 3).

Participants for all experiments were recruited through Louisiana State University’s human-participant study pool and provided written informed consent. The experiments were approved by the university’s institutional review board. Sample sizes were determined on the basis of previous research involving fear conditioning and mental imagery (Greening et al., 2022).

## Experiment 1

### Method

#### Participants

Participant data showing no detectable SCR to the mild electrical stimulation (i.e., *nonresponders*) and poor recordings resulting from excessive movement or technical failure were removed from both the SCR and self-report data analyses (Lonsdorf et al., 2017). We identified poor recordings by visual inspection after filtering the time-series data but before segmenting them into condition-level epochs. Poor recordings were those in which high-frequency noise, large spikes, and/or sudden drops in signal meant that we would be unable to reliably discriminate between noise and an SCR response, even to the US. For full transparency, we provide our analyses of the self-report data on the full sample of participants, including those who were removed, in the Supplemental Material available online.

Forty-five healthy adults (38 women) between the ages of 18 and 32 years (*M* = 20.3) successfully completed the experiment. The data of 12 participants were removed: Six were nonresponders to the US, and six had excessive noise in their SCR data. The final sample included 33 participants.

#### Materials

During the visual-acquisition phase (described in more detail below), rightward-oriented Gabor patches (45° angle producing lines from the lower left to upper right corner) and horizontal Gabor patches (0° angle producing lines from left to right) were used as the CS+ and CS−. The CS+ and CS− assignment was counterbalanced across participants. Participants viewed and imagined both the CS+ and CS− throughout this phase. Stimuli were presented within 8° of visual angle of fixation, which was at the center of the screen. Trial-by-trial auditory instructions—“imagine right,” “imagine horizontal,” “attend right,” and “attend horizontal”—were created using an online app (www.fromtexttospeech.com) and were presented using a standard stereo speaker mounted to the monitor. Prior to completing the phase, participants received instructions on the stimuli and task (see Section 1.1.1 in the Supplemental Material).

During the imagery-acquisition phase, the CS+ and CS− were leftward-oriented Gabor patches (135° angle from horizontal, producing lines from lower right to upper left corner) and vertically oriented Gabor patches (90° angle from horizontal, producing lines from bottom to top), counterbalanced across participants. The instructions were “imagine left,” “imagine vertical,” “attend left,” and “attend vertical,” which were created and presented in the same manner as described for the visual-acquisition phase; this included instructions prior to completion of the phase (see Section 1.1.2 in the Supplemental Material).

Mild electrical stimulation (administered using the STMISOC and STM100C modules, BIOPAC, Goleta, CA) served as the US. Two electrodes were placed on the fingertips of the index and middle fingers of the participant’s nondominant hand. The stimulation duration was 2 ms. Each participant set the intensity of shock to a level that was deemed “uncomfortable but not painful,” consistent with previous research (*M* = 5.23 mA; Jiang et al., 2021; Schiller et al., 2010).

#### Procedure and design

Experiment 1 consisted of two phases: the visual-acquisition phase and the imagery-acquisition phase. The order in which the phases were administered was counterbalanced across participants. Each phase began with one habituation run that was identical to the task itself but did not include trials with the US. After the habituation run, participants completed four runs of a differential-fear-conditioning-acquisition task. At the end of each phase, the participants completed a Likert-style questionnaire associated with the phase (see Sections 1.4 and 1.5 in the Supplemental Material).

The overall trial structure was identical for both the visual-acquisition and imagery-acquisition phases. Each trial began with a black fixation dot for 1.5 s while the auditory instruction was presented. The black dot remained on the screen for an additional 4 s while the participants either viewed or imagined the Gabor patch, as indicated in the auditory instruction. The fixation dot then turned white for a 12-s intertrial interval. Prior to the experiment, participants were instructed that when the fixation dot turned black, they should listen to and follow the instructions and that when the fixation dot turned white, they should prepare for the next trial. Prior to the start of each acquisition phase, participants were told there was a chance of receiving a shock; however, they were never instructed as to which CS would be paired with a shock.

##### Visual-acquisition phase

**Fig. 1.**
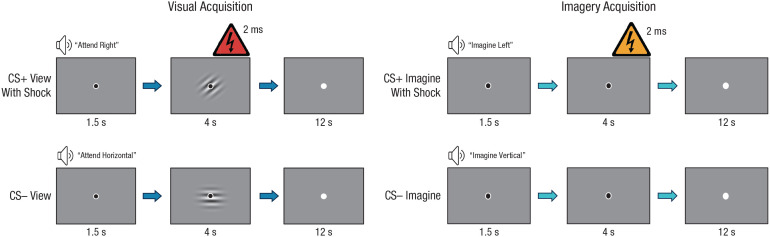
Example trial structures in the visual-acquisition and imagery-acquisition phases in Experiment 1. Each trial began with an auditory cue instructing participants to either attend to (i.e., view) or imagine the conditioned stimulus (CS), which was a Gabor patch tilted horizontally, vertically, to the right, or to the left. Next, participants viewed or imagined the cued Gabor patch for 4 s. Disappearance of the black fixation dot signaled the intertrial interval. Trials in which participants were instructed to view or imagine the CS+ or CS– were presented with a 2-ms mild electrical stimulation (top row) and without stimulation (bottom row).

In the visual-acquisition phase, if the auditory instruction indicated that the participant should view the CS+ and the trial included the US, the relevant Gabor patch and the black fixation dot were presented in the center of the screen for 4 s and the participant received mild electrical stimulation for 2 ms that ended at the same time as the Gabor patch disappeared. If the trial did not include the US, participants viewed the relevant CS+ or CS− Gabor patch and black fixation dot for 4 s. Trials on which participants were instructed to imagine the CS+ or CS– followed this same structure; however, the black fixation dot appeared on the screen with no patch for 4 s while the participant imagined the patch (see Fig. 1).

Participants completed four runs, and each run consisted of 12 trials: two view trials with a CS+ and a shock, two view trials with a CS+ but no shock, four view trials with a CS−, two imagine trials with a CS+, and two imagine trials with a CS−. The trial order was pseudorandom. Each run began and ended with a trial in which participants viewed the CS−, which was excluded from the analyses (Grégoire & Greening, 2020). In the first run of the phase, participants viewed the CS+ with shock in the second trial. The other trial in which participants viewed the CS+ with shock was randomly presented within the second half of the run. For the remaining three runs, the first trial in which participants viewed the CS+ with shock was randomly presented in the first half of the run, and the second was randomly presented in the second half of the run. All other trials not otherwise noted were randomly assigned within the run.

##### Imagery-acquisition phase

The design of the imagery-acquisition phase was identical to that of the visual-acquisition phase; however, participants received a mild electrical stimulation when imagining the CS+ rather than when viewing the CS+. When presented, the mild electrical stimulation ended at the same time that participants were signaled to stop imagining the CS+, as in the visual-acquisition phase (Fig. 1).

Each run included 12 trials during the imagery-acquisition phase as well: two imagine trials with a CS+ and a shock, two imagine trials with a CS+ but no shock, four imagine trials with a CS−, two view trials with a CS+, and two view trials with a CS−. Again, the trial order was pseudorandom. Each run began and ended with trial in which participants imagined the CS−, which was excluded from analyses. In the first run of the phase, participants imagined the CS+ with shock in the second trial. The other trial in which participants imagined the CS+ with shock was randomly presented within the second half of the run. For the remaining three runs, the first trial in which participants imagined the CS+ with shock was randomly presented in the first half of the run, and the second trial was randomly presented in the second half of the run. All other trials not otherwise noted were randomly assigned within the run.

#### Self-report assessment

After each phase, participants completed a Likert-style, self-reported questionnaire that evaluated (a) their fear of getting shocked in each type of trial (1, *not at all*, to 7, *very much so*; e.g., “How much did you fear the shock on VIEW LEFT trials?”), (b) the vividness of the mental images on imagine trials (1, *none existent*, to 7, *very strong*; e.g., “How vivid was your mental imagery on IMAGINE LEFT trials?”), and (c) the effort needed to form the mental images on imagine trials (1, *not at all*, to 7, *very hard*; e.g., “How hard did you try to form the mental images on IMAGINE LEFT trials?”). For each question, participants were asked specifically about one of the two Gabor patches. Moreover, to avoid biasing participants, we made no reference to “CS+” or “CS−” (see Sections 1.4 and 1.5 in the Supplemental Material).

Participants also completed the Vividness of Visual Imagery Questionnaire (Marks, 1973), the State-Trait Anxiety Inventory (Spielberger et al., 1983), and the Attentional Control Scale (Derryberry & Reed, 2002) for potential secondary post hoc analyses (see Section 2.1 in the Supplemental Material). Two-tailed analyses are reported for all results unless otherwise specified.

#### Skin conductance monitoring

As in previous research, SCRs were recorded at 2000-Hz sampling rates with the MP150 system (BIOPAC; Jiang et al., 2021). Two Ag/AgCl electrodes with a conductive saline-based gel (BIOPAC GEL101) were placed on the fingertips of the ring and pinky fingers of the nondominant hand. The SCR analyses were conducted in MATLAB (Version 2017a; The MathWorks, Natick, MA). The whole time series was preprocessed with a band-pass filter employing a first-order Butterworth filter and cutoff frequencies of 5 Hz and 0.01 Hz, respectively (Bush et al., 2018). This helps remove low-frequency drift and spurious high-frequency noise from the data (Bach et al., 2009). Finally, the size of the time-series signal was reduced to 100 Hz.

SCRs were calculated by subtracting baseline (average signal between 0 s and 1 s) from the maximum peak amplitude during the 1- to 6-s time window following the onset of the CS (Greening et al., 2016; Jiang et al., 2021). Each SCR was also required to be greater than 0.02 µS. If these criteria were not met, the SCR for the trial was scored as zero. The trials that included shocks were excluded from analyses because the SCRs on these trials could be potentially confounded with the unconditioned response and not the conditioned fear response (Jiang et al., 2021). The first and last trials were always CS− trials, which we also excluded from analyses to control for the possibility that novelty effects during the first trial would create spurious SCRs, as well as to ensure an identical number of trials for each condition (Grégoire & Greening, 2020). SCRs were square-root transformed to attain normality prior to statistical analyses. Two-tailed analyses are reported for all results unless otherwise specified.

#### Bayesian analysis

For each critical analysis presented below, we also include a two-sided Bayesian analysis by computing the Bayes factor (BF) for the likelihood that the alternative hypothesis (H_1_; the presence of an effect) is true relative to the likelihood that the null hypothesis (H_0_; no effect) is true (BF_10_) or vice versa (BF_01_). The prior distribution of H_1_ was derived from a Cauchy distribution with a scaling factor of 0.707 (Krypotos et al., 2017). To evaluate how our choice of prior distribution might have contributed to our analysis, we also conducted a sensitivity analysis using Cauchy distributions with the scaling factors of 0.707, 1, and 1.41 (Krypotos et al., 2017). The strength of evidence for BF_10_ or BF_01_ changing across different priors is not uncommon; however, the sensitivity analysis allowed us to evaluate the reliability by investigating whether the direction of BF_10_ varied in directionality (e.g., was it consistently greater than 1).

### Results

#### Self-report ratings of fear

**Fig. 2.**
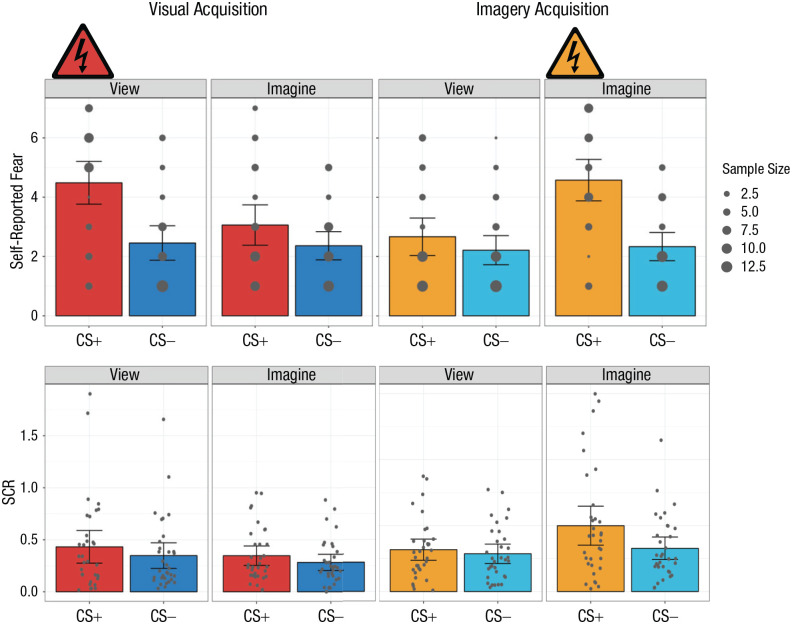
Mean self-reported fear (top row) and skin conductance response (SCR; bottom row) in the visual-acquisition and imagery-acquisition phases of Experiment 1. Results are shown separately for each conditioned stimulus (CS) type (CS+ and CS−) and percept modality (view CS and imagine CS) in each phase. Self-reported fear was rated on a scale from 1, *not at all*, to 7, *very much so*. The size of each dot in the top row indicates how many participants chose that value. In the bottom row, each dot represents a participant’s mean SCR for a given condition, and each data bar represents a group mean. For all graphs, error bars represent 95% confidence intervals. The shock symbol indicates the condition in which participants were fear conditioned in each acquisition phase.

A 2 × 2 × 2 repeated measures analysis of variance (ANOVA) with the factors CS type (CS+ or CS−), percept modality (view or imagine), and phase (visual acquisition or imagery acquisition) was conducted (Fig. 2; see Section 2.2.1 in the Supplemental Material). There was a significant three-way interaction, *F*(1, 32) = 24.52, *p* < .001, η^2^ = .05. There was also a significant interaction between percept modality and phase, *F*(1, 32) = 16.38, *p* < .001, η^2^ = .07. Finally, there was a significant main effect of CS type, *F*(1, 32) = 36.21, *p* < .001, η^2^ = .14. No other statistically significant results were found (see Section 2.2.2 in the Supplemental Material). Importantly, in the supplemental analysis, the results of inferential tests on self-reported fear data in the full sample were identical (see Section 2.2.3 in the Supplemental Material). Below, we unpack the critical three-way interaction in a manner related initially to our three primary hypotheses.

In the visual-acquisition phase, participants reported significantly more fear when viewing the CS+ compared with viewing the CS−, *t*(32) = 4.48, *p* < .001, *d* = 0.78. Importantly, there was significant generalization of differential conditioning when participants imagined the CS+ compared with when they imagined the CS−, *t*(32) = 2.23, *p* = .033, *d* = 0.39.

In the imagery-acquisition phase, participants had significantly greater self-reported fear when imagining the CS+ than imagining the CS−, *t*(32) = 6.44, *p* < .001, *d* = 1.12. The acquisition of differential conditioning also appeared to generalize to viewing the CS, as revealed by greater self-reported fear when participants viewed the CS+ than when they viewed the CS−, *t*(32) = 3.14, *p* = .004, *d* = 0.55.

To further understand the three-way interaction, we used follow-up *t* tests comparing various conditions. Notably, within the visual-acquisition phase, the viewed CS+ was significantly greater than the imagined CS+, *t*(32) = 3.15, *p* = .004, *d* = 0.55. Conversely, in the imagery-acquisition phase, the imagined CS+ was significantly greater than the viewed CS+, *t*(32) = 4.42, *p* < .001, *d* = 0.77. Comparing the two phases, we found significantly greater self-reported fear when participants were conditioned to the viewed CS+ (visual-acquisition phase) than when they generalized to the viewed CS+ (imagery-acquisition phase), *t*(32) = 4.11, *p* < .001, *d* = 0.72. Similarly, there was significantly greater fear reported when participants were conditioned to the imagined CS+ (imagery-acquisition phase) than when they generalized to the imagined CS+ (visual-acquisition phase), *t*(32) = 4.10, *p* < .001, *d* = 0.71. All other comparisons were nonsignificant (see Section 2.2.4 in the Supplemental Material).

In the visual-acquisition phase, the Bayesian analysis assessing participants’ self-reported fear when viewing the CS+ versus viewing the CS− resulted in very strong, decisive evidence for H_1_, BF_10_(0.707) = 280.8. Evaluation of the generalization of differential conditioning when participants imagined the CS+ compared with when they imagined the CS− resulted in anecdotal evidence for H_1_ relative to H_0_, BF_10_(0.707) = 1.62.

In the imagery-acquisition phase, the Bayesian analysis assessing the participants’ fear when imagining the CS+ and imagining the CS− resulted in very strong, decisive evidence for H_1_ relative to H_0_, BF_10_(0.707) > 1,000.00. Assessing participants’ fear when viewing the CS+ and viewing the CS− resulted in strong evidence for H_1_, BF_10_(0.707) = 10.35. See Section 2.2.5 in the Supplemental Material for a sensitivity analysis of each Bayesian result reported above.

#### Self-report ratings of imagery effort and vividness

To evaluate differences in self-reported imagery vividness and effort between conditions, we ran two independent 2 × 2 ANOVAs using the factors CS type (CS+ or CS−) and phase (visual acquisition or imagery acquisition) for each. These analyses revealed no significant findings (see Sections 2.3.1 and 2.4.1 in the Supplemental Material). Regarding important descriptive statistics relating to the participants’ subjective sense of vivid imagery, the median rating was 5 (range = 2–7; for a frequency graph, see Section 2.3.2 in the Supplemental Material) on the Likert-style questionnaire (1, *none existent*, to 7, *very strong*). Regarding participants’ imagery effort, the median rating was 5 (range = 2–7; for a frequency graph, see Section 2.4.2 in the Supplemental Material) on the Likert-style questionnaire (1, *not at all*, to 7, *very hard*).

#### Skin conductance response

A 2 × 2 × 2 repeated measures ANOVA with the factors CS type (CS+ or CS−), percept modality (view or imagine), and phase (visual acquisition or imagery acquisition) was also conducted (see Section 2.5.1 in the Supplemental Material). There was a significant three-way interaction, *F*(1, 32) = 5.59, *p* = .024, η^2^ = .004. There was also a significant interaction between percept modality and phase, *F*(1, 32) = 10.79, *p* = .002, η^2^ = .02. The interaction between CS type and percept modality was also significant, *F*(1, 32) = 4.89, *p* = .03, η^2^ = .002. Finally, there was a significant main effect of CS type, *F*(1, 32) = 9.10, *p* = .005, η^2^ = .02. No other statistically significant results were found (see Fig. 2, bottom row; see also Section 2.5.2 in the Supplemental Material). To unpack the three-way interaction, we proceeded as described below, starting with comparisons most relevant to our primary hypotheses.

In the visual-acquisition phase, we observed a significantly larger SCR when participants viewed the CS+ than when they viewed the CS−, *t*(32) = 2.21, *p* = .034, *d* = 0.38. Importantly, we also found a significant generalization effect—there was a larger SCR when participants imagined the CS+ than when they imagined the CS−, *t*(32) = 2.33, *p* = .026, *d* = 0.41.

Notably, in the imagery-acquisition phase, we observed a significantly greater SCR when participants imagined the CS+ than when they imagined the CS−, *t*(32) = 3.18, *p* = .003, *d* = 0.55. Conversely, regarding generalization, there was no significant difference in SCR when participants viewed the CS+ than when they viewed the CS−, *t*(32) = 0.89, *p* = .382, *d* = 0.15. However, we ran a post hoc follow-up analysis using a one-tailed *t* test (Schiller et al., 2010) to investigate generalization from imagining to viewing, using only participants who showed evidence of differential conditioning (i.e., CS+ minus CS− > 0) on imagine trials. This analysis revealed significantly greater SCR when participants viewed the CS+ than when they viewed the CS−, consistent with generalization in this subsample of participants (*n* = 22), *t*(21) = 1.81, *p* = .042, one-tailed, *d* = 0.38.

We used follow-up *t* tests to further understand the three-way interaction. Within the imagery-acquisition phase, the SCR to the imagined CS+ was significantly greater than to the viewed CS+, *t*(32) = 3.98, *p* < .001, *d* = 0.69. Between phases, the conditioned response to the imagined CS+ in the imagery-acquisition phase was significantly greater than the generalization response to the imagined CS+ in the visual-acquisition phase, *t*(32) = 2.65, *p* = .01, *d* = 0.46. On the other hand, the generalization effect to the viewed CS+ from the visual-acquisition phase was not significantly greater than the generalization effect to the viewed CS+ from the imagery-acquisition phase, *t*(32) = 2.03, *p* = .051, *d* = 0.35, though this effect might indicate a trend toward significance. All other comparisons were nonsignificant (see Section 2.5.3 in the Supplemental Material).

##### Bayesian analysis of SCR data

In the visual-acquisition phase, the Bayesian analysis of the SCR data revealed that viewing the CS+ versus viewing the CS− resulted in anecdotal evidence for H_1_ relative to H_0_, BF_10_(0.707) = 1.56. Assessing participants’ SCR when imagining the CS+ versus imagining the CS− also resulted in anecdotal evidence for H_1_ relative to H_0_, BF_10_(0.707) = 1.94.

In the imagery-acquisition phase, assessing the participants’ SCR when imagining the CS+ versus imagining the CS− resulted in strong evidence for H_1_ relative to H_0_, BF_10_(0.707) = 11.30. Last, the analysis of participants’ fear when viewing the CS+ and viewing the CS− resulted in substantial evidence for H_0_ relative to H_1_, BF_01_(0.707) = 3.74.

See Section 2.5.4 in the Supplemental Material for a sensitivity analysis of each Bayesian result above.

##### Temporal SCR analyses

**Fig. 3.**
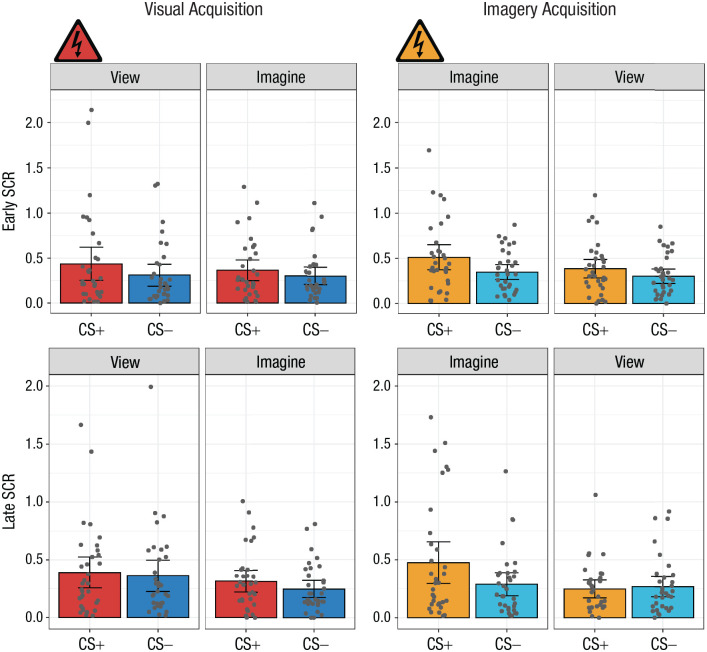
Mean skin conductance response (SCR) in the early (first two runs; top row) and late (second two runs; bottom row) stages of the visual-acquisition and imagery-acquisition phases of Experiment 1. Results are shown separately for each conditioned stimulus (CS) type (CS+ and CS−) and percept modality (view CS and imagine CS) in each phase. Each dot represents a participant’s mean SCR for a given condition, and each data bar represents a group mean. Error bars represent 95% confidence intervals. The shock symbol indicates the condition in which participants were fear conditioned in each acquisition phase.

In addition to the primary analyses, we evaluated whether the effects observed above were a function of acquisition timing (i.e., early- vs. late-acquisition trials). A 2 × 2 × 2 repeated measures ANOVA with the factors CS type (CS+ or CS−), percept modality (view or imagine), and time (early or late, defined by the first two runs and the second two runs of a phase, respectively) was conducted independently for each acquisition phase (Fig. 3). In the visual-acquisition phase, only the main effect of CS was found, *F*(1, 32) = 6.77, *p* = .014, η^2^ = .01 (see Section 2.5.5 in the Supplemental Material). In the imagery-acquisition phase, the analysis revealed a significant interaction of CS type and percept modality, *F*(1, 32) = 6.40, *p* = .017, η^2^ = .01. It also revealed a significant main effect of time, *F*(1, 32) = 7.26, *p* = .011, η^2^ = .01; a main effect of CS type, *F*(1, 32) = 6.31, *p* = .017, η^2^ = .03; and a main effect of percept modality, *F*(1, 32) = 10.00, *p* = .003, η^2^ = .03 (see Section 2.5.6 in the Supplemental Material). No additional analyses were conducted.

## Experiment 2

The auditory cue at the beginning of both visual and imagery trials in Experiment 1 could have been responsible for the observed generalization effects. In Experiment 2, we replicated the visual-acquisition phase from Experiment 1 but ruled out the possibility that participants were being conditioned to the auditory cue. To do so, we retained the auditory cues on imagery trials but excluded them from visual trials.

### Method

#### Participants

Experiment 2 included only the visual-acquisition phase described in Experiment 1 (Fig. 1). Thirty-four healthy adults (28 women) between the ages of 18 and 36 years (*M* = 20.6) successfully completed the experiment. The data of seven participants were removed: Four were nonresponders (no detectable response after receiving a shock), and recording was poor for another three (too much noise resulting from technical failure or excessive participant movement in the SCR data). Thus, data from 27 participants were used in analyses. For descriptive statistics from the additional self-reported questionnaires, see Section 3.1 in the Supplemental Material.

#### Materials

All materials in Experiment 2 were identical to those in Experiment 1 (mild electrical stimulation; *M* = 3.49 mA).

#### Procedure and design

Experiment 2 was identical to the visual-acquisition phase in Experiment 1 except for the auditory cues. In Experiment 2, the auditory instructions were presented only prior to imagine trials, similar to the visual-acquisition phase in Experiment 1. Prior to view trials, however, no auditory instruction was presented, and the appropriate stimulus appeared on the screen with no cue. This also shortened the trial duration for view trials because they did not include the 1.5-s black fixation point for the instruction, as indicated in the instructions given to participants prior to the experiment (see Section 1.2 in the Supplemental Material).

#### Skin conductance monitoring and self-report assessment

Skin conductance monitoring and self-report assessment were conducted as in Experiment 1.

### Results

#### Subjective ratings

**Fig. 4.**
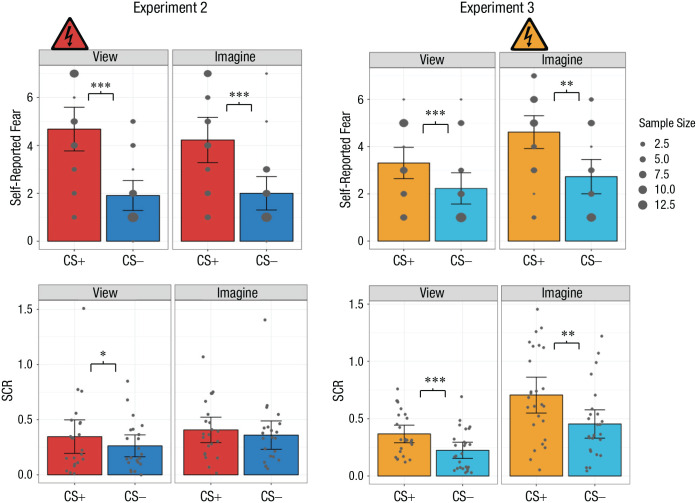
Mean self-reported fear (top row) and skin conductance response (SCR; bottom row) in Experiments 2 and 3. Results are shown separately for each conditioned stimulus (CS) type (CS+ and CS−) and percept modality (view CS and imagine CS) in each experiment. Self-reported fear was rated on a scale from 1, *not at all*, to 7, *very much so*. The size of each dot in the top row indicates how many participants chose that value. In the bottom row, each dot represents a participant’s mean SCR for a given condition, and each data bar represents a group mean. For all graphs, error bars represent 95% confidence intervals. The shock symbol indicates the condition in which participants were fear conditioned in each acquisition phase. Asterisks indicate significant differences between responses to each CS type (*p < .05, **p < .01, ***p < .001), as determined via *t* tests.

A 2 × 2 repeated measures ANOVA with the factors CS type (CS+ or CS−) and percept modality (view or imagine) was conducted. The interaction between CS type and percept modality was significant, *F*(1, 26) = 17.91, *p* < .037, η^2^ = .01. There was also a significant main effect of CS type, *F*(1, 26) = 34.19, *p* < .001, η^2^ = .30. There was no main effect of percept modality (Fig. 4, top left; see also Sections 3.2.1 and 3.2.2 in the Supplemental Material). Importantly, in the supplemental analysis, the self-reported-fear results from the full sample supported the same inferences (see Section 3.2.3 in the Supplemental Material).

Unpacking the interaction, we found that participants reported significantly more fear when viewing the CS+ than when viewing the CS−, *t*(26) = 5.95, *p* < .001, *d* = 1.14. Critically, participants also self-reported greater fear when imagining the CS+ than when imagining the CS−, *t*(26) = 4.91, *p* < .001, *d* = 0.94. No other significant pairwise differences were observed (see Section 3.2.4 in the Supplemental Material).

#### Bayesian analysis of self-reported fear data

The Bayesian analysis assessing participants’ fear when viewing the CS+ and viewing the CS− resulted in very strong, decisive evidence for H_1_ relative to H_0_, BF_10_(0.707) = 6806.17. Likewise, assessing participants’ generalized fear when imagining the CS+ and imagining the CS− resulted in very strong, decisive evidence for H_1_ relative to H_0_, BF_10_(0.707) = 568.90. For the corresponding sensitivity analyses, see Section 3.2.5 in the Supplemental Material.

#### Self-report ratings of imagery effort and vividness

Two *t* tests were also conducted to examine whether there were differences between stimuli (CS+ or CS−) in self-reported effort or vividness. The test investigating effort (range = 2–7, *Mdn* = 6) was not significant, *t*(26) = 1.28, *p* = .21, *d* = 0.25; however, self-reported vividness was significantly greater when imagining the CS+ (range = 3–7, *Mdn* = 6) than the CS− (range = 2–7, *Mdn* = 5), *t*(26) = 2.18, *p* = .04, *d* = 0.42 (for a frequency graph, see Section 3.2.6 in the Supplemental Material).

#### Skin conductance response

A 2 × 2 ANOVA with the factors CS type (CS+ or CS−) and percept modality (view or imagine) was conducted. The interaction between CS type and percept modality was not significant. However, there was a significant main effect of CS type, *F*(1, 26) = 5.24, *p* = .03, η^2^ = .01; irrespective of percept modality, SCR was greater to the CS+ (*M* = 0.35, *SD* = 0.27) than the CS− (*M* = 0.29, *SD* = 0.23), *t*(26) = 2.29, *p* = .03, *d* = 0.44. There was also a significant main effect of percept modality, *F*(1, 26) = 7.37, *p* = .01, η^2^ = .02; irrespective of CS type, SCR was greater during imagine trials (*M* = 0.37, *SD* = 0.25) than view trials (*M* = 0.28, *SD* = 0.25), *t*(26) = 2.72, *p* = .01, *d* = 0.52 (Fig. 4, bottom left; see also Sections 3.3.1 and 3.3.2 in the Supplemental Material).

Despite finding no interaction, we conducted two follow-up *t* tests to investigate one of our primary hypotheses. Participants had larger SCRs to the viewed CS+ than the viewed CS−, *t*(26) = 2.13, *p* = .04, *d* = 0.41. However, the difference between imagining the CS+ and imagining the CS− was not significant, *t*(26) = 1.03, *p* = .31, *d* = 0.20.

##### Bayesian analysis of SCR data

The Bayesian analysis assessing participants’ SCR when viewing the CS+ and viewing the CS− resulted in anecdotal evidence for H_1_ relative to H_0_, BF_10_(0.707) = 1.41. Assessing participants’ fear when imagining the CS+ and imagining the CS− resulted in evidence for H_0_ relative to H_1_, BF_01_(0.707) = 3.04. In investigating the trend of CSs irrespective of perception modality, we found anecdotal evidence for H_1_ relative to H_0_, BF_10_(0.707) = 1.86. For sensitivity analysis of each Bayesian analysis, see Section 3.3.3 in the Supplemental Material.

##### Temporal SCR analyses

**Fig. 5.**
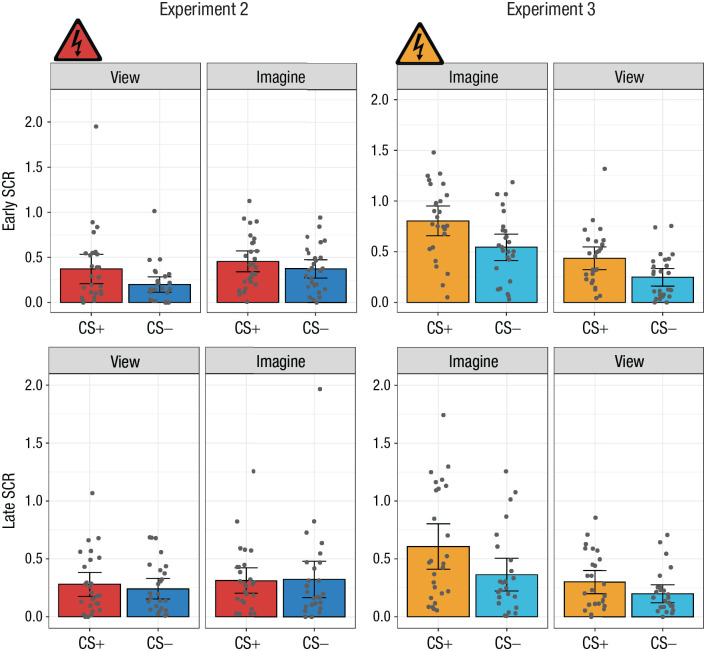
Mean skin conductance response (SCR) in the early (first two runs; top row) and late (second two runs; bottom row) stages of Experiments 2 and 3. Results are shown separately for each conditioned stimulus (CS) type (CS+ and CS−) and percept modality (view CS and imagine CS) in each experiment. Each dot represents a participant’s mean SCR for a given condition, and each data bar represents a group mean. Error bars represent 95% confidence intervals. The shock symbol indicates the condition in which participants were fear conditioned in each acquisition phase.

A 2 × 2 × 2 repeated measures ANOVA with the factors CS type (CS+ or CS−), percept modality (view or imagine), and time (early or late, defined by the first two runs and the second two runs of the study, respectively) was conducted (Fig. 5; see also Section 3.3.4 in the Supplemental Material). It reported a significant main effect of time, *F*(1, 26) = 5.51, *p* = .027, η^2^ = .01; a main effect of CS type, *F*(1, 26) = 6.28, *p* = .019, η^2^ = .01; and a main effect of percept modality, *F*(1, 26) = 11.48, *p* = .002, η^2^ = .02.

## Experiment 3

We designed Experiment 3 to replicate and extend the imagery-acquisition phase from Experiment 1 but to rule out the possibility that participants were conditioned to the auditory cue. To do so, we retained the auditory cues on imagery trials but excluded them from visual trials.

### Method

#### Participants

Experiment 3 included only the imagery-acquisition phase (Fig. 1). Thirty-two 32 healthy adults (26 women) between the ages of 18 and 24 years (*M* = 20.13) successfully completed the experiment. The data of six participants were removed: Two were nonresponders (no detectable response after receiving a shock), and four had too much noise in their SCR data because of poor recordings. Thus, data from 26 participants were used in analyses (for descriptive statistics from the supplementary questionnaires, see Section 4.1 in the Supplemental Material).

#### Materials

All materials in Experiment 3 were identical to those in Experiment 1 (mild electrical stimulation; *M* = 6.68 mA).

#### Procedure and design

Experiment 3 was identical to the imagery-acquisition phase in Experiment 1, except that the same adjustments were made to the auditory cue as in Experiment 2. In Experiment 3, the auditory instructions were presented prior to each imagery trial, as in Experiment 1, except that when participants viewed a Gabor patch, no auditory instruction was presented. During these trials, the appropriate patch appeared on the screen with no cue. This also shortened the intertrial interval for trials in which participants viewed the patch, because they did not include the 1.5-s fixation point for the instruction. The intertrial interval for trials in which participants imagined the patch remained identical to those in the imagery-acquisition phase in Experiment 1 (see Section 1.3 in the Supplemental Material). Skin conductance monitoring and self-report assessment were conducted as in Experiment 1.

### Results

#### Subjective ratings

A 2 × 2 ANOVA with the factors CS type (CS+ or CS−) and percept modality (view or imagine) was conducted. The interaction between CS type and percept modality was significant, *F*(1, 25) = 5.89, *p* = .023, η^2^ = .01. There was also a significant main effect of CS type, *F*(1, 25) = 16.72, *p* < .001, η^2^ = .16. There was also a significant main effect of percept modality, *F*(1, 25) = 19.66, *p* < .001, η^2^ = .07 (Fig. 4, top right; see also Sections 4.2.1 and 4.2.2 in the Supplemental Material). In the supplemental analysis, the self-reported-fear results from the full sample supported the same inferences (see Section 4.2.3 in the Supplemental Material).

Further interrogating the interaction, we observed that participants reported significantly greater fear in response to the imagined CS+ than the imagined CS−, which indicates differential conditioning, *t*(25) = 4.00, *p* < .001, *d* = 0.78. Furthermore, we observed significant generalization of the conditioned response: Fear was greater for the viewed CS+ than the viewed CS−, *t*(25) = 3.49, *p* = .002, *d* = 0.68.

We also compared responses to each CS type. Regarding the CS+, imagining the CS+ produced greater fear than viewing the CS+, *t*(25) = 4.12, *p* < .001, *d* = 0.81. Imagining the CS− also produced significantly greater fear than viewing the CS−, *t*(25) = 2.58, *p* = .02, *d* = 0.51.

#### Bayesian analysis self-reported fear data

Assessing participants’ fear when imagining the CS+ and imagining the CS− using Bayesian analysis resulted in strong evidence for H_1_ relative to H_0_, BF_10_(0.707) = 63.18. Assessing participants’ fear when viewing the CS+ and viewing the CS− resulted in strong, decisive evidence for H_1_ relative to H_0_, BF_10_(0.707) = 20.51. For sensitivity analyses for each of the above analyses, see Section 4.2.4 in the Supplemental Material.

#### Self-report ratings of imagery effort and vividness

Two *t* tests were also conducted to examine whether there were differences between stimuli (CS+ or CS−) in self-reported effort or vividness. The test investigating effort (range = 1–7, *Mdn* = 6 across conditions) was not significant, *t*(25) = 0.34, *p* = .74, *d* = 0.07, nor was the test investigating self-reported vividness (range = 2–7, *Mdn* = 5.5 across conditions) when participants imagined the CS+ than when they imagined the CS−, *t*(25) = 0.13, *p* = .90, *d* = 0.03 (for frequency graphs, see Section 4.2.5 in the Supplemental Material).

#### Skin conductance response

A 2 × 2 ANOVA with the factors CS type (CS+ or CS−) and percept modality (view or imagine) was again conducted. The interaction between CS type and percept modality was significant, *F*(1, 25) = 4.77, *p* = .039, η^2^ = .01. There was also a significant main effect of CS type, *F*(1, 25) = 25.76, *p* < .001, η^2^ = .12, and a significant main effect of percept modality, *F*(1, 25) = 33.52, *p* < .001, η^2^ = .21 (Fig. 4, bottom right; see also Sections 4.3.1 and 4.3.2 in the Supplemental Material).

Follow-up *t* tests revealed a significantly greater SCR to the imagined CS+ than the imagined CS−, *t*(25) = 4.80, *p* < .001, *d* = 0.94. Similarly, we observed significant generalization of fear conditioning, as indicated by larger SCRs to the viewed CS+ than the viewed CS−, *t*(25) = 3.67, *p* = .001, *d* = 0.72.

Comparing CS types, we also observed significant differences. Imagining the CS+ produced greater SCRs than viewing the CS+, *t*(25) = 5.61, *p* < .001, *d* = 1.10. Imagining the CS− also produced significantly greater SCRs than viewing the CS−, *t*(25) = 4.67, *p* < .001, *d* = 0.92.

##### Bayesian analysis of SCR data

Using Bayesian analysis to assess participants’ differential SCRs when imagining the CS+ and imagining the CS−, we found very strong, decisive evidence for H_1_ relative to H_0_, BF_10_(0.707) = 409.45. Regarding the generalized SCR, viewing the CS+ versus viewing the CS− resulted in strong evidence for H_1_ relative to H_0_, BF_10_(0.707) = 30.32, BF_01_(0.707) = 0.03. For sensitivity analyses, see Section 4.3.3 in the Supplemental Material.

##### Temporal SCR analyses

A 2 × 2 × 2 repeated measures ANOVA with the factors CS type (CS+ or CS−), percept modality (view or imagine), and time (early or late, defined by the first two runs and the second two runs of the study, respectively) was conducted (Fig. 5; see also Section 4.3.4 in the Supplemental Material). This revealed a significant interaction of CS type and percept modality, *F*(1, 32) = 4.77, *p* = .036, η^2^ = .01; a significant main effect of time, *F*(1, 32) = 12.05, *p* = .002, η^2^ = .05; a main effect of CS type, *F*(1, 32) = 25.76, *p* < .001, η^2^ = .09; and a main effect of percept modality, *F*(1, 32) = 33.52, *p* < .001, η^2^ = .17. Given the lack of an interaction involving time, we performed no additional analyses.

## Discussion

The current study demonstrates that mental imagery of basic visual objects affects the processes of differential fear conditioning. Three experiments combining fear conditioning with mental imagery and visual perception revealed evidence for our three hypotheses: (a) Differential fear conditioning acquired to visual percepts generalizes to matching imagined percepts, (b) differential fear conditioning can be established to imagined percepts, and (c) differential fear conditioning acquired to imagined percepts generalizes to matching visual percepts.

Regarding our first hypothesis, that differential fear acquired to the visual percepts generalizes to the matching imagined percepts, self-reported fear and SCRs from the visual-acquisition phase of Experiments 1 and 2 are consistent with fear conditioning to visual percepts generalizing to imagined percepts. According to both null-hypothesis significance testing (NHST) and Bayesian analyses, participants self-reported significant differential fear conditioning for imagined percepts in the visual-acquisition phase in Experiments 1 and 2. SCRs in the visual-acquisition phase of Experiment 1 also showed differential fear conditioning to imagined percepts. When investigating SCRs in Experiment 2, we observed a main effect: SCRs were greater to the CS+ than the CS−. The present study replicates the findings of Greening et al. (2022), demonstrating that differential fear conditioning acquired to visual percepts generalizes to the corresponding imagined percepts. This finding is consistent with research showing that imagery of the CS can contribute to fear extinction (Agren et al., 2017; Jiang & Greening, 2021; Reddan et al., 2018) and fear reconsolidation (Grégoire & Greening, 2019). It is also notable that the present results indicate that imagery can be used to express generalized differential fear conditioning without a consolidation period following acquisition.

Consistent with our second hypothesis, that differential fear conditioning can be acquired to imagined percepts, the imagery-acquisition phase in Experiments 1 and 3 revealed that differential fear conditioning can be acquired to imagined percepts. In both experiments, using both NHST and Bayesian analysis, we found that self-reported levels of fear and SCRs indicated robust differential fear conditioning to a mentally imagined stimulus when paired with a US. The Bayesian analysis indicated that these are the largest effects in the study across both self-reported fear and SCR measures. These results are consistent with the earlier findings of Holzman and Levis (1991). However, the present study also addressed many of the limitations noted in the introduction regarding Holzman and Levis (1991), including the need to evaluate differential conditioning.

The final hypothesis concerned whether differential fear acquired to imagined percepts generalizes to the corresponding visual percepts. Support was found in both NHST and Bayesian analysis of the self-reported levels of fear collected in the imagery-acquisition phase in Experiments 1 and 3. In the imagery-acquisition phase of Experiment 1, both NHST and Bayesian analysis of SCRs showed generalization of differential fear conditioning to a viewed stimulus but only in participants who showed successful differential fear acquired to the imagined percepts. The SCR results of Experiment 3 provided support for the generalization of differential fear to the visual percepts in both NHST and Bayesian analysis. The results from Experiments 1 and 3 indicate that following differential fear conditioning to imagined percepts, there is evidence of differential fear generalization to the corresponding visual percepts without a need for a consolidation period following acquisition. These results are consistent with the evaluative-conditioning results of Lewis et al. (2013), who found that viewing CSs after the imagined CSs were paired with negative and positive images produced a significant priming effect to congruent versus incongruent emotional images.

Though not a primary aim of this study, an interesting and novel finding concerns the strength of fear for the percept modality paired with shock. The inclusion of both phases in Experiment 1 allowed us to compare the differential conditioning of visual percepts with the differential conditioning of imagined stimuli. In self-reported levels of fear and SCR measures, the acquisition of fear to each percept modality showed no significant difference. The strength of response to the CS+ of the percept modality paired with shock was significantly greater than the response to the CS+ of the percept modality not paired with shock (e.g., during imagery acquisition, the response to imagining the CS+ was greater than the response to viewing the CS+). In both self-reported levels of fear and SCR, the generalization of fear to the percept modality not paired with the US also showed no significant difference. These findings show that the conditioned response to the CS+ was significantly higher to the percept modality that was paired with shock, and fear conditioning to an imagined versus a viewed CS+ did not produce a difference in the conditioned-response magnitude.

Our study also revealed noteworthy effect-size differences between the self-reported fear effects and the SCR effects, in particular relating to the generalization results. One potential explanation is that measures of conscious cognition versus measures of physiology such as SCR are measuring at least partially dissociable aspects of fear conditioning (Clark & Squire, 1998). On the other hand, recent research indicates that self-reported fear and physiological measures of fear such as SCR are correlated (Jiang et al., 2021), though differential-conditioning effects appear larger for self-report measures compared with SCRs, potentially because of differences in measurement error. Future research is required to further clarify the relationship between different measures of differential fear conditioning and whether or to what extent they measure dissociable constructs.

Along with other recent research involving mental imagery and fear (Hoppe et al., 2022; Koizumi et al., 2017; Reddan et al., 2018; Wicken et al., 2021), we interpret our findings as being consistent with the depictive theory, indicating that mental images activate early perceptual representations containing some geometric information of the physical stimulus without the stimulus being physically present (Pearson et al., 2015), and consistent with experience-dependent associative mechanisms (Johansen et al., 2011). For example, during imagery acquisition (Experiments 1 and 3), imagery of the CSs activates a neural representation in early visual regions, similar to the representation generated when one views the CS, leading to the subsequent activation of brain regions associated with the expression of fear conditioning such as the amygdala (Braem et al., 2017; Greening et al., 2022). This interpretation is consistent with our findings of robust self-reported imagery vividness and with previous research showing that imagery vividness is associated with early visual cortex functioning (Albers et al., 2013; Bergmann et al., 2016; Harrison & Tong, 2009). Specifically, we found that across all three experiments, all participants rated their vividness as greater than 1 (on a scale from 1, *none existent*, to 7, *very strong*), and the median rating was 5 or greater across all imagery conditions. However, several alternative explanations of the present findings ought to be considered.

The auditory cues prior to every trial in Experiment 1 may account for the generalization effects. This is unlikely because participants reported generating mental images of the Gabor patches that were subjectively vivid and that were associated with fear of receiving the shock. Thus, participants were not passively listening to the auditory cue but did visualize the instructed imagery. Finally, results of inferential tests on the generalization effect in Experiments 2 and 3, in which the auditory cues on visual trials were removed, revealed similar findings to those of Experiment 1.

Another alternative is that participants formed propositional (i.e., descriptive) knowledge about the relationship between the CSs and the US (Mitchell et al., 2009), as in semantic generalization (Boyle et al., 2016; Grégoire & Greening, 2020) or instructed fear conditioning (Mechias et al., 2010). Yet it is unlikely that generalization from imagery acquisition to viewing the Gabor patches, or vice versa, is due to similar semantic elements. For example, fear conditioning to object names does not appear to generalize to the corresponding visual objects or vice versa (Branca, 1957), nor does fear conditioning to visual objects generalize to semantically related sounds, as measured with SCRs (Gerdes et al., 2020). On the other hand, imagery acquisition (e.g., Experiment 3) might involve implicit instructed fear conditioning in which one descriptively learns the association between the Gabor patches and the US, thereby leading to generalized conditioned responses when one views the CSs. This explanation cannot entirely be ruled out, though it may itself require mental imagery, as depictive mental imagery may underpin instructed fear conditioning (Olsson & Phelps, 2007). Nevertheless, our findings, along with those reported in previous literature, suggest that differential conditioning and generalization to imagined CS can occur, although the underlying mechanisms—be they depictive (Greening et al., 2022; Pearson et al., 2015), descriptive (Mertens et al., 2020; Mitchell et al., 2009), or some combination of both—remain up for debate (Ji et al., 2016). Future research on how the internal representations of mental images are stored and how they affect differential conditioning, potentially using brain imaging, is needed.

It should be noted that the stimuli and sample used in this study may have limited the generalizability of our findings. Whereas the present study used low-complexity visual objects (i.e., Gabor patches) as CSs, previous research indicates that lower complexity objects are on average more vividly imagined that higher complexity objects (Dijsktra et al., 2017). On the other hand, a recent study of fear extinction indicates that object complexity is not a critical factor of imagery extinction (Hoppe et al., 2022). Another consideration is that object imagery appears dissociable from spatial imagery (Kozhevnikov et al., 2005; Blazhenkova, 2016). Future research is required to evaluate how our findings would generalize to objects of higher complexity or imagery of spatial stimuli. The present study also relied on a random sample of college-age younger adults. Previous research indicates that there may be age-group differences in imagery vividness and emotion (Murphy et al, 2017; Heyes et al. 2013), which might produce age-group differences in our observed effects. In addition, other research indicates that differences in imagery vividness might be present in people with mental illness (Holmes et al., 2008; Matthews et al., 2014). Future research is required to evaluate whether the observed effects in the present study would generalize to alternative populations such as those who are younger or older or those with a mental disorder.

## Supplemental Material

sj-docx-1-pss-10.1177_09567976221086513 – Supplemental material for Fear in the Theater of the Mind: Differential Fear Conditioning With Imagined Stimuli Supplemental material, sj-docx-1-pss-10.1177_09567976221086513 for Fear in the Theater of the Mind: Differential Fear Conditioning With Imagined Stimuli by Lauryn Burleigh, Xinrui Jiang and Steven G. Greening in Psychological Science

## Transparency

*Action Editor:* Daniela Schiller

*Editor:* Patricia J. Bauer

*Author Contributions*

S. G. Greening developed the study concept. L. Burleigh and S. G. Greening designed the study. Testing and data collection were performed by L. Burleigh. L. Burleigh analyzed and interpreted the data under the supervision of S. G. Greening. L. Burleigh and S. G. Greening drafted the manuscript with help from X. Jiang, and X. Jiang provided critical revisions. All the authors approved the final manuscript for submission.

**Open Practices:** All data, analysis code, and code used to generate the stimuli have been made publicly available via OSF and can be accessed at https://osf.io/zdc8t/. The design and analysis plans for the experiments were not preregistered. This article has received the badges for Open Data and Open Materials. More information about the Open Practices badges can be found at http://www.psychologicalscience.org/publications/badges.
