# Supplementary material for: Fear in the Theater of the Mind: Differential Fear Conditioning With Imagined Stimuli
Source: Psychol Sci. 2022 Jul 27;33(9):1423–39. doi: 10.1177/09567976221086513 (PMC13021123; doi:10.1177/09567976221086513)
Supplement: sj-docx-1-pss-10.1177_09567976221086513 – Supplemental material for Fear in the Theater of the Mind: Differential Fear Conditioning With Imagined Stimuli [file sj-docx-1-pss-10.1177_09567976221086513.docx]

## Supplemental

### 1. Methods

#### 1.1 Instructions given prior to Experiment 1

##### 1.1.1 Visual Acquisition phase instructions

#####
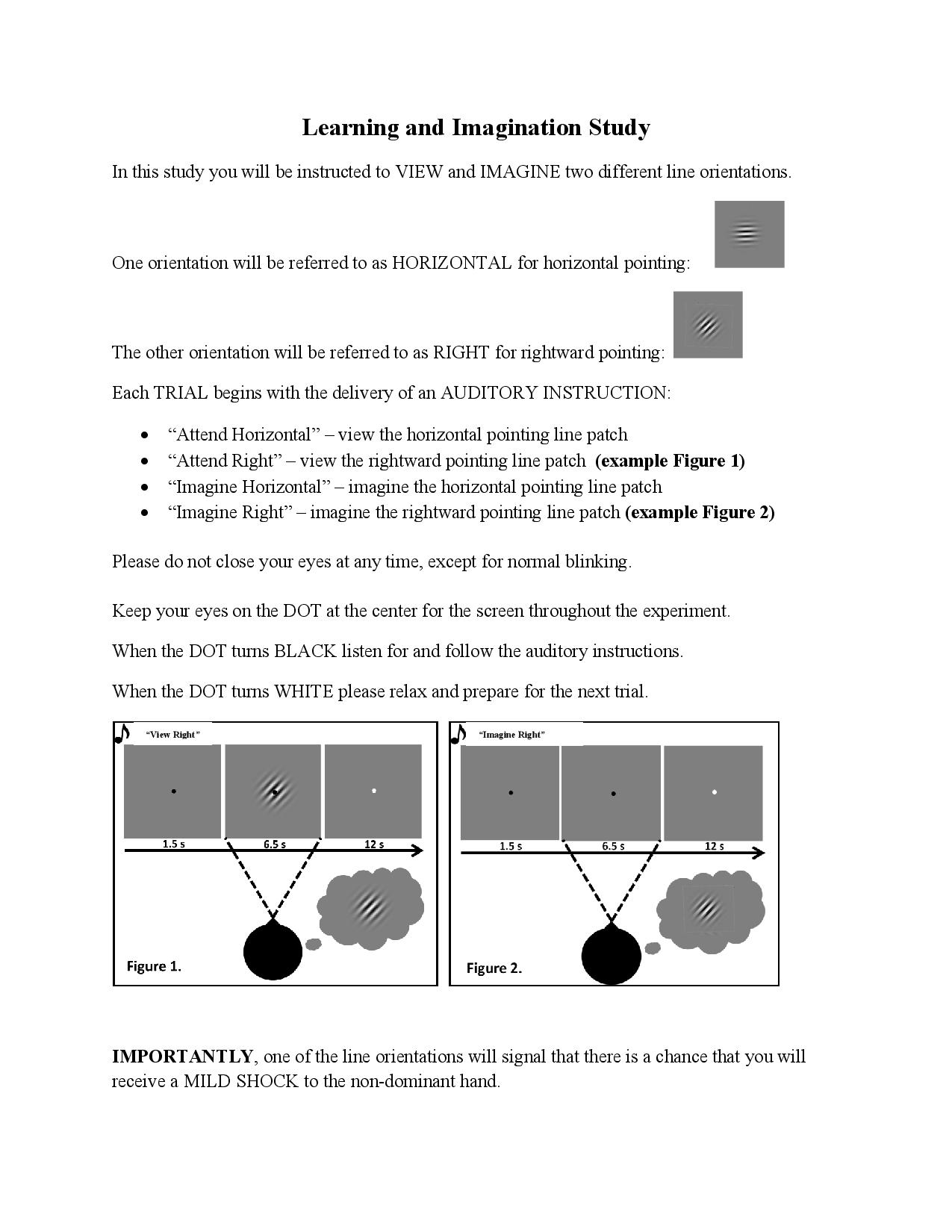


Fig. S1. Each participant was guided through these instructions prior to receiving the Visual Acquisition phase in Experiment 1.

##### 1.1.2 Imagery Acquisition phase instructions


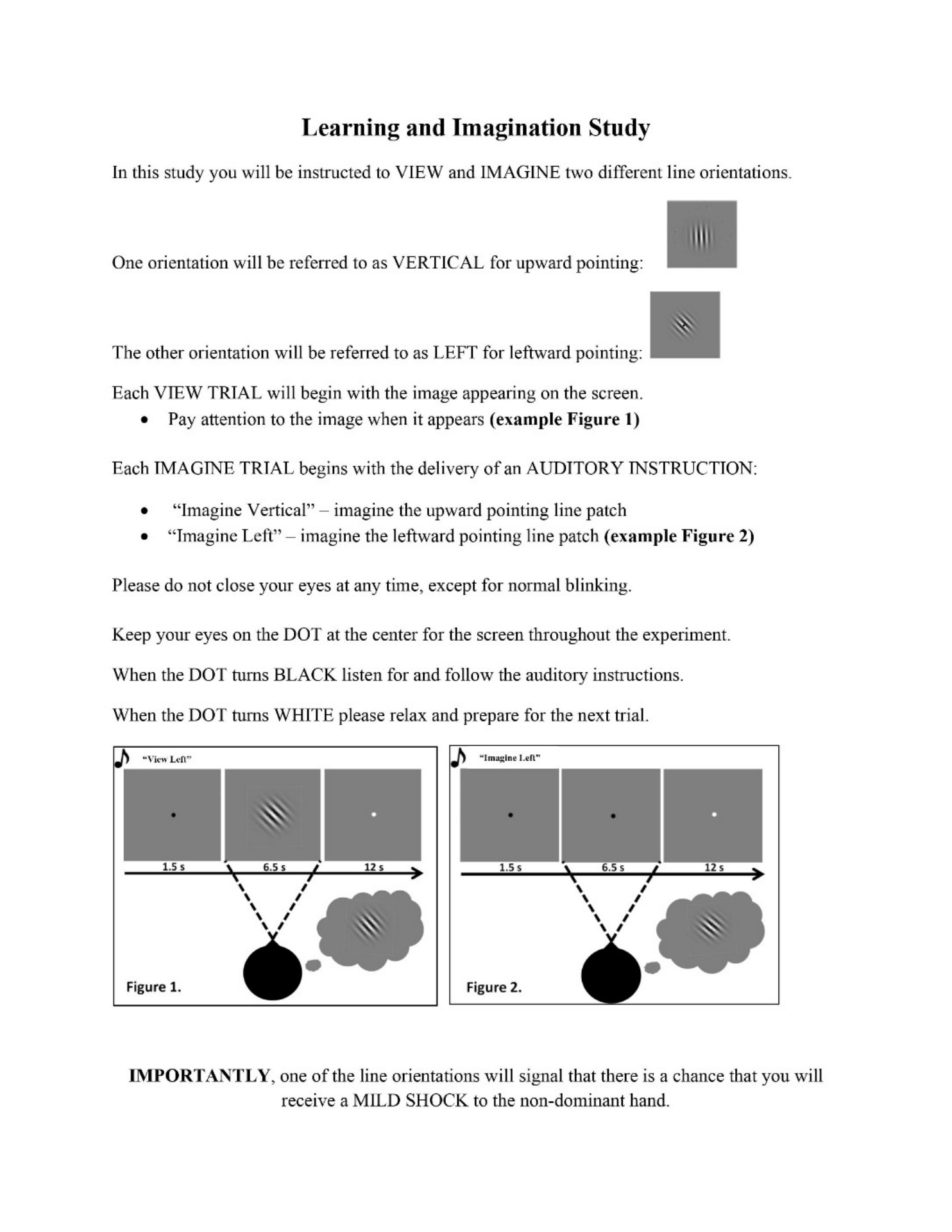


Fig. S2. Each participant was guided through these instructions prior to receiving the Imagery Acquisition phase in Experiment 1.

#### 1.2 Instructions given prior to Experiment 2


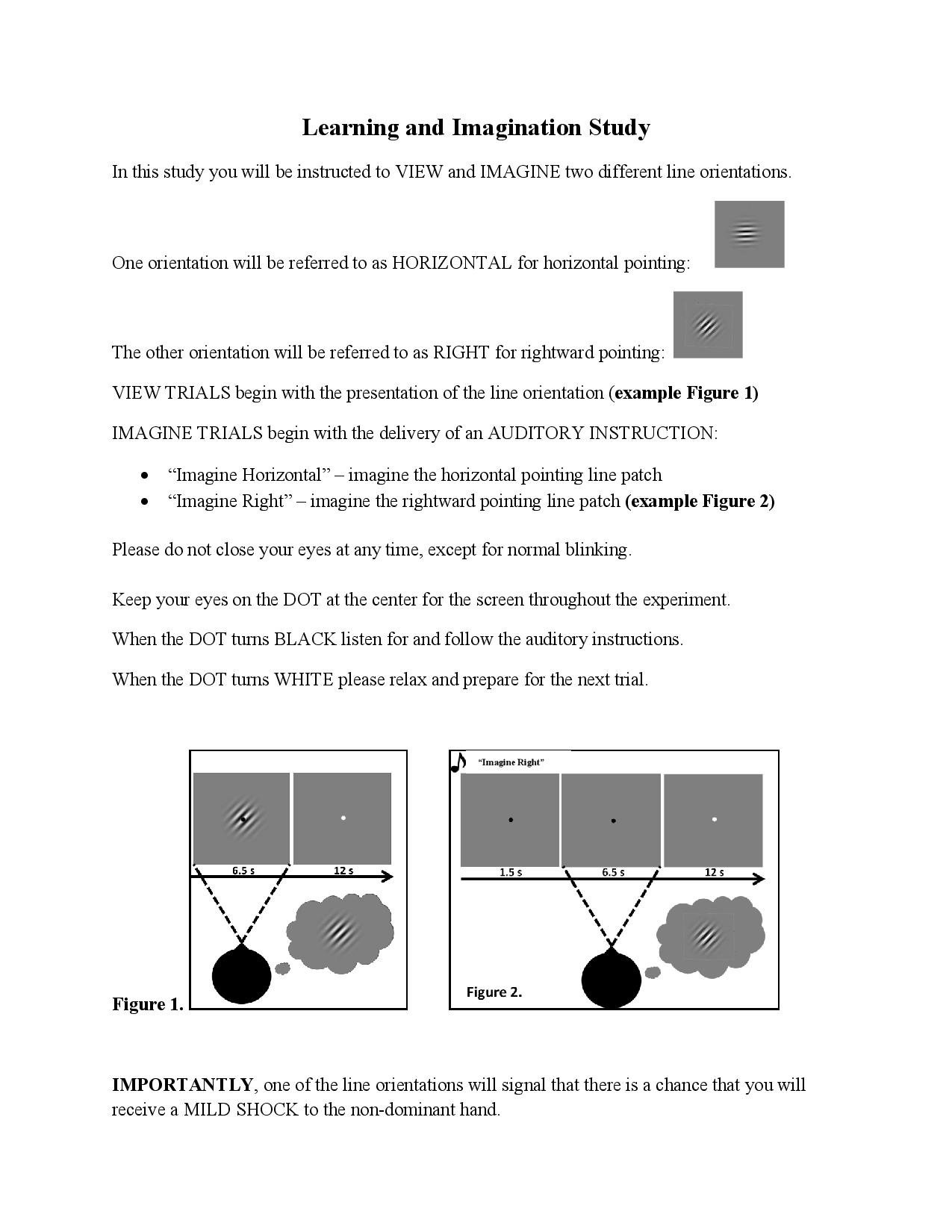


Fig. S3. Each participant was guided through these instructions prior to Experiment 2.

#### 1.3 Instructions given prior to Experiment 3


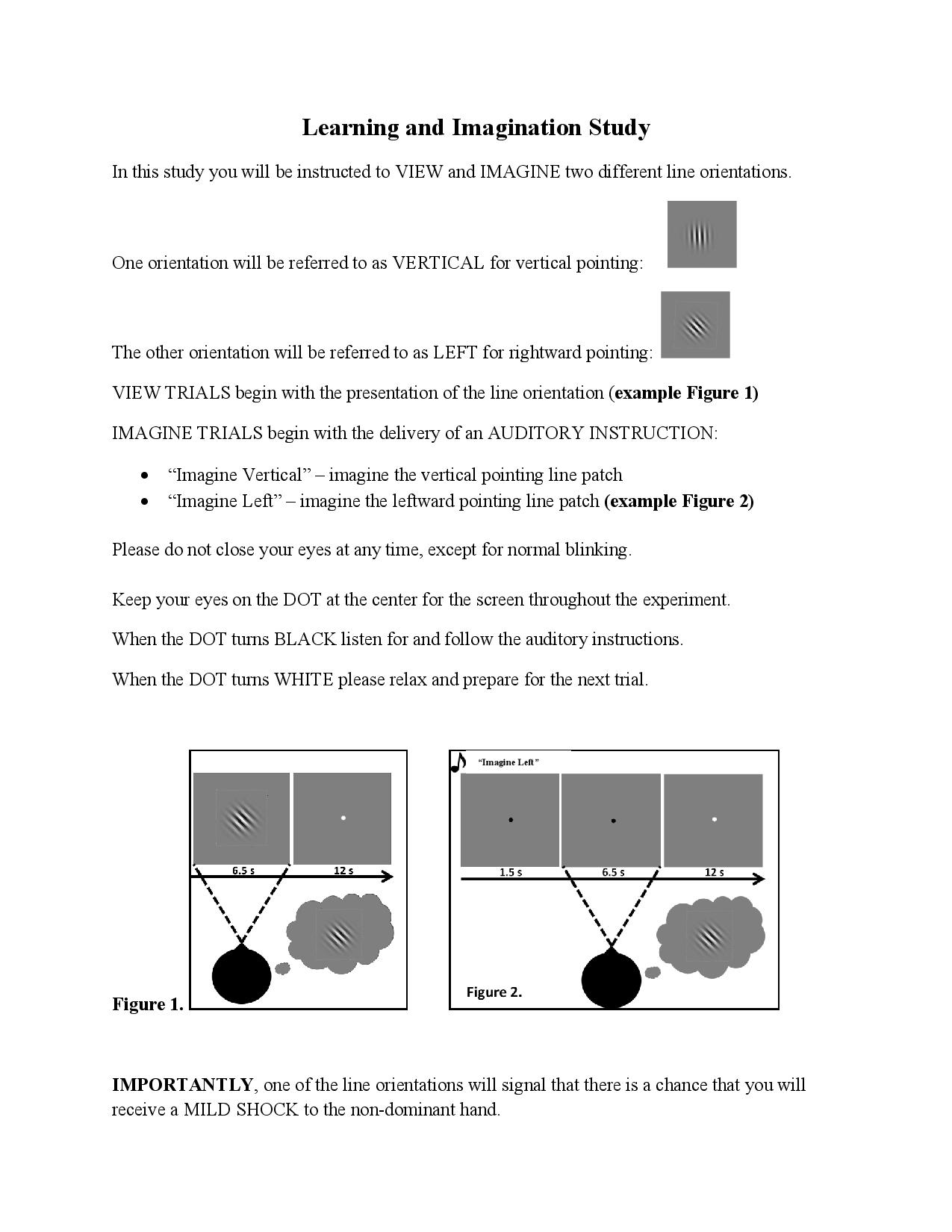


Fig. S4. Each participant was guided through these instructions prior to Experiment 3.

#### 1.4 Likert-Style Questionnaire Visual Acquisition

####
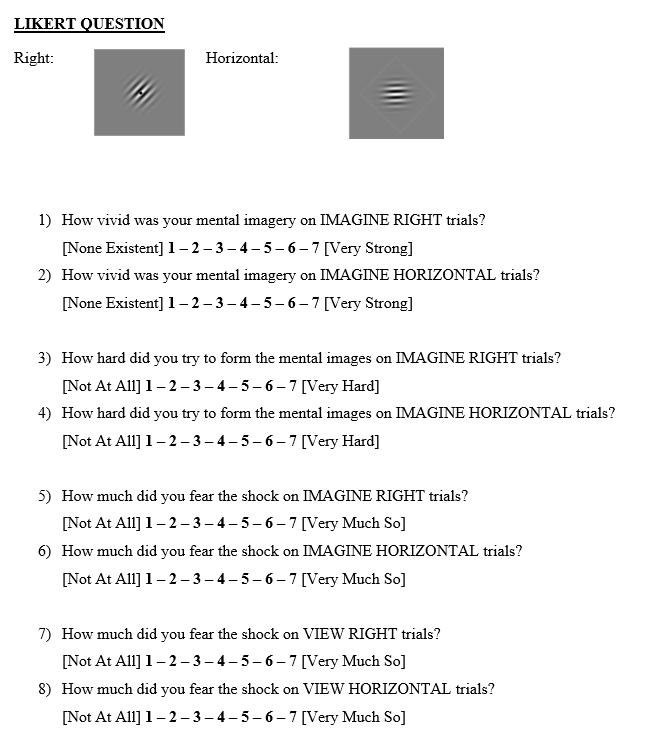


Fig. S5. Each participant answered this self-reported questionnaire after receiving the Visual Acquisition phase.

#### 1.5 Likert-Style Questionnaire Imagery Acquisition


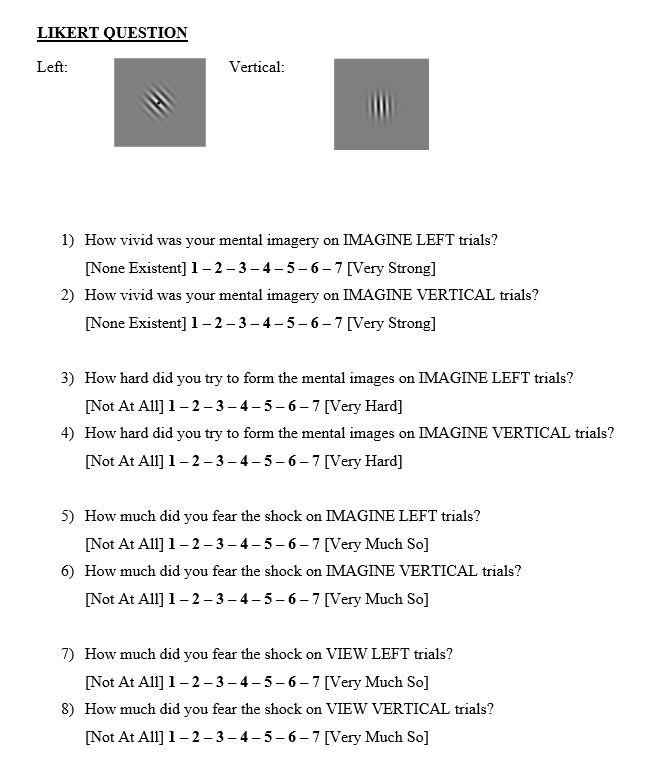


Fig. S6. Each participant answered this self-reported questionnaire after receiving the Imagery Acquisition phase.

### 2. Experiment 1 Results

#### 2.1 Subjective Questionnaires

|  | Mean | SD |
| --- | --- | --- |
| VVIQ | 62.61 | 10.55 |
| State Anxiety Inventory | 46.86 | 4.74 |
| Trait Anxiety Inventory | 39.53 | 11.44 |
| Attentional Control Scale | 49.53 | 8.51 |

Table S1. Descriptive statistics of questionnaires completed by participants in Experiment 1

#### 2.2 Self-Reported Fear

##### 2.2.1 Likert-style Descriptive Data

|  | Visul Acquisition Phase | | Imagery Acquisition Phase | |
| --- | --- | --- | --- | --- |
|  | Mean | SD | Mean | SD |
| Vividness: CS+ imagine | 4.97 | 1.45 | 4.85 | 1.61 |
| Vividness: CS- imagine | 5.03 | 1.24 | 5.06 | 1.37 |
| Effort: CS+ imagine | 4.91 | 1.68 | 4.91 | 1.68 |
| Effort: CS- imagine | 4.82 | 1.45 | 4.88 | 1.47 |
| Fear: CS+ view | 4.48 | 2.03 | 2.67 | 1.78 |
| Fear: CS- view | 2.45 | 1.64 | 2.21 | 1.39 |
| Fear: CS+ imagine | 3.06 | 1.92 | 4.58 | 1.97 |
| Fear: CS- imagine | 2.36 | 1.34 | 2.33 | 1.34 |

Table S2. Descriptive statistics of self-reported, likert-style questionnaire completed for each phase by participants in Experiment 1

#####

##### 2.2.2 Self-Reported Fear ANOVA Results


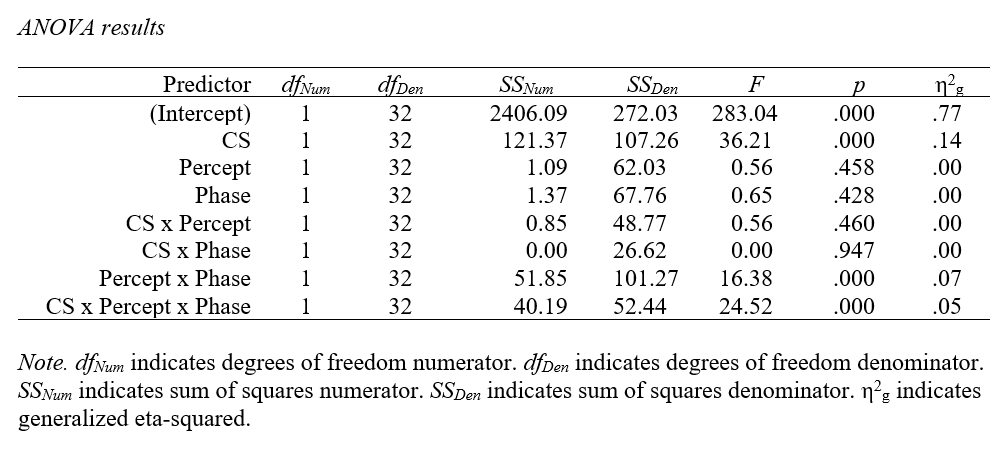


Table S3. Full table of ANOVA results using the self-reported data from Experiment 1. The 2x2x2 ANOVA consisted of CS (CS+ vs CS-), Percept (viewed stimulus vs imagined stimulus), and Phase (Visual Acquisition Phase vs Visual Acquisition Phase)

##### 2.2.3 Self-Reported Fear ANOVA Results with All Participants


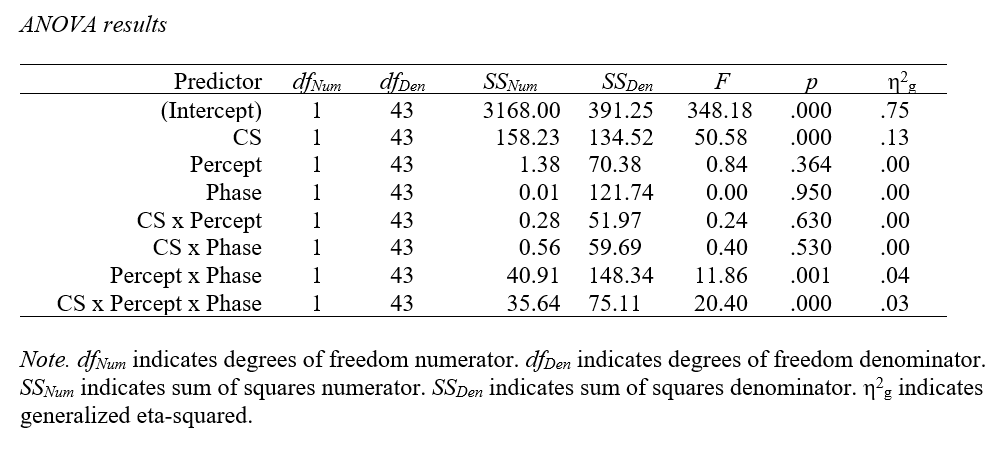


Table S4. Full table of ANOVA results using the self-reported data from all participants in Experiment 1. This table includes participants who’s SCRs were deemed as too noisy or non-responders. The 2x2x2 ANOVA consisted of CS (CS+ vs CS-), Imagery (viewed stimulus vs imagined stimulus), and Set (Visual Acquisition Phase vs Visual Acquisition Phase)

##### 2.2.4 Self-Reported Fear Pairwise Comparison Results

In order to unpack the three-way interaction we conducted pairwise comparisons between the various conditions. Here we report those results that are not included in the main manuscript, as they are all non-significant.

There was no significant difference between conditioning to the viewed CS+ (Visual Acquisition phase) and conditioning to the imagined CS+ (Imagery Acquisition phase), *t*(32) = .27, *p* = .786. There was also no significant difference between the generalized percepts, irrespective of the acquisition phase. The imagined CS+ from the Visual Acquisition phase was not different from the visual CS+ from the Imagery Acquisition phase, *t*(32) = 1.11, *p* = .277.

Lastly, we evaluated the CS- conditions’ potential contributions to the three-way interaction and found no significant pairwise difference between the various CS- conditions. In the Visual Acquisition phase there was no significant difference between the imagined CS- and the viewed CS-, *t*(32) = .36, *p* = .72. In the Imagery Acquisition phase there was also no difference between the imagined CS- and viewed CS-, *t*(32) = .56, *p* = .580. Across the two phases, there was no significantly different self-reported fear when conditioning to a viewed CS- (Visual Acquisition phase) versus generalizing to a viewed CS- (Imagery Acquisition phase), *t*(32) = 0.76, *p* = 0.45. Similarly, there was no significantly different fear reported when participants were conditioned to an imagined CS- (Imagery Acquisition phase) versus generalizing to an imagined CS- (Visual Acquisition phase), *t*(32) = 0.16, *p* = 0.88. Moreover, there was no significant difference between conditioning to a visual CS- (Visual Acquisition phase) and conditioning to an imagined CS- (Imagery Acquisition phase), *t*(32) = 0.43, *p* = 0.67. There was also no significant difference between the generalized percepts irrespective of the acquisition phase. The imagined CS- from the Visual Acquisition phase was not different from the visual CS- from the Imagery Acquisition phase, *t*(32) = 0.56, *p* = 0.58.

##### 2.2.5 Bayesian Analysis Self-Reported Fear data

In the Visual Acquisition phase, the Bayesian Sensitivity Analysis assessing the participants’ self-reported fear when viewing the CS+ versus viewing the CS- indicates that this conclusion is likely very reliable, BF_10_(0.707) = 280.80, BF_10_(1) = 280.80, and BF_10_(1.41) = 230.72. Evaluation of the generalization of differential conditioning when imagining the CS+ compared to imagining the CS- resulted a sensitivity analysis of BF_10_(0.707) = 1.62, BF_10_(1) = 1.29, and BF_10_(1.41) = 0.98, indicating modest reliability but that the direction of the effect could be affected by the choice of the prior.

In the Imagery Acquisition phase, the Bayesian Sensitivity Analysis assessing the participants’ fear when imagining the CS+ and imagining the CS- resulted in: BF_10_(0.707) = 52548.20, BF_10_(1) = 56488.58, and BF_10_(1.41) = 54555.19. The Sensitivity Analysis assessing the participants’ fear when viewing the CS+ and viewing the CS- resulted in: BF_10_(0.707) = 10.35, BF_10_(1) = 8.85, and BF_10_(1.41) = 7.07.

#### 2.3 Vividness Ratings

##### 2.3.1 Vividness ANOVA

#####
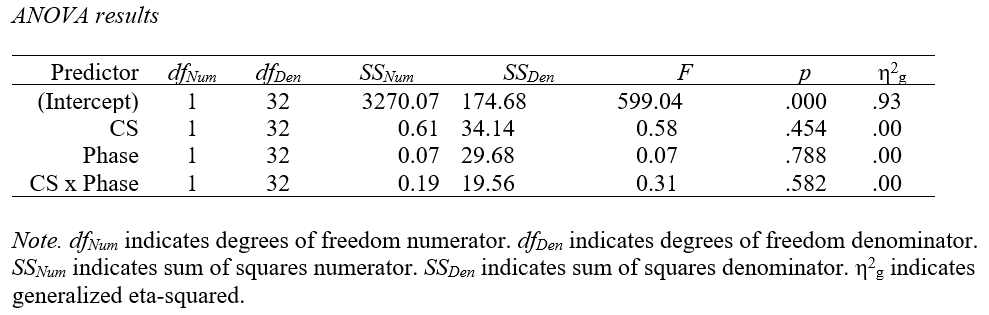


Table S5. Full table of ANOVA results using the vividness data from Experiment 1. The 2x2 ANOVA consisted of CS (CS+ vs CS-) and Phase (Visual Acquisition Phase vs Visual Acquisition Phase)

##### 2.3.2 Vividness Frequency Graph


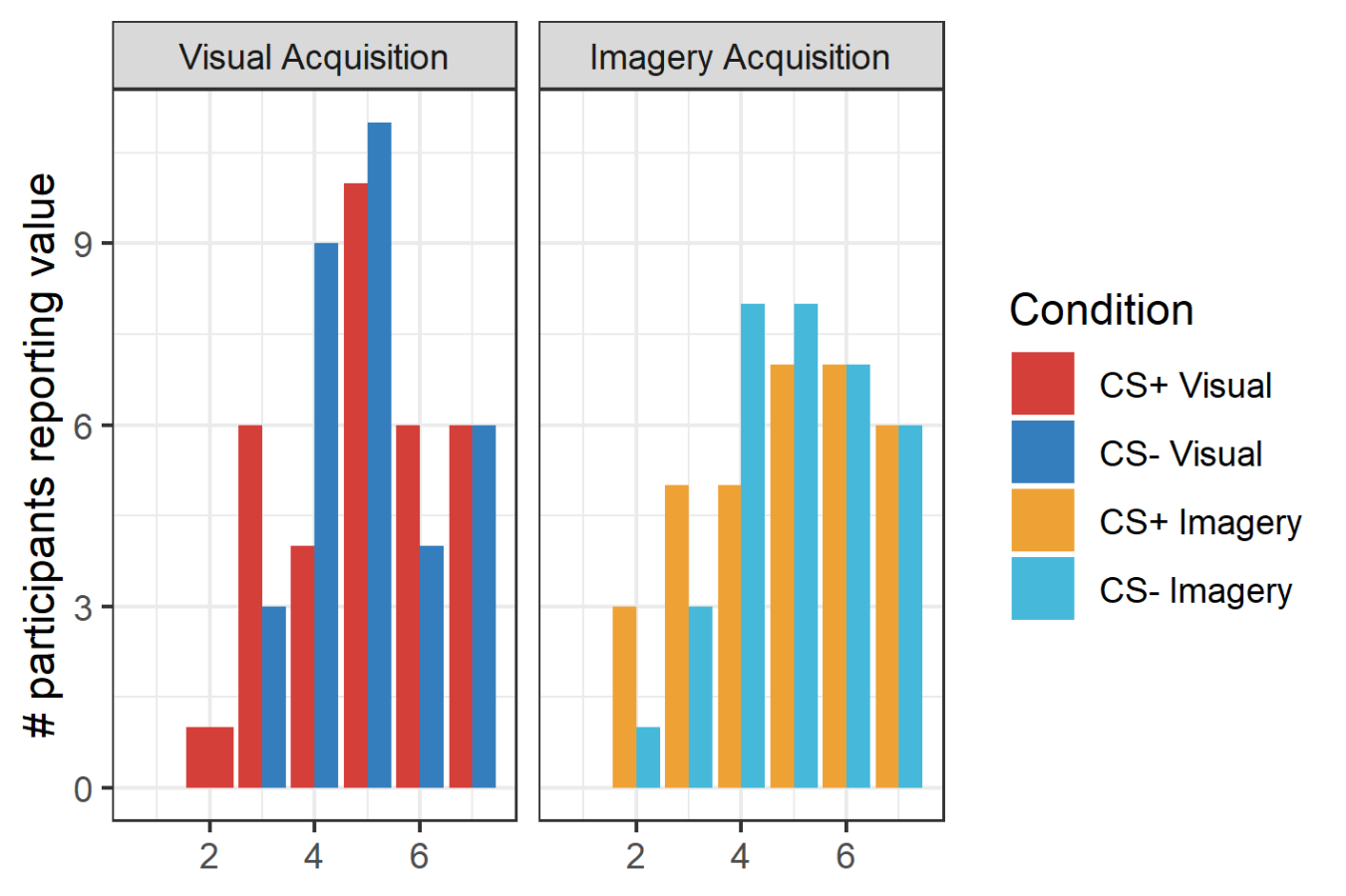


Fig. S7. Each bar represents the number of participants that reported the given value (1 (None Existent – 7 (Very Strong)) on the Likert-Style Questionnaire regarding vividness for each condition in Experiment 1. Red and dark blue bars represent imagining the CS+ and CS-, respectively, in the Visual Acquisition phase while orange and light blue bars represent imagining the CS+ and CS-, respectively, in the Imagery Acquisition phase. Note that the presence of a double wide bar indicates that that value was only chosen for one of the two conditions.

#### 2.4 Effort Ratings

##### 2.4.1 Effort ANOVA


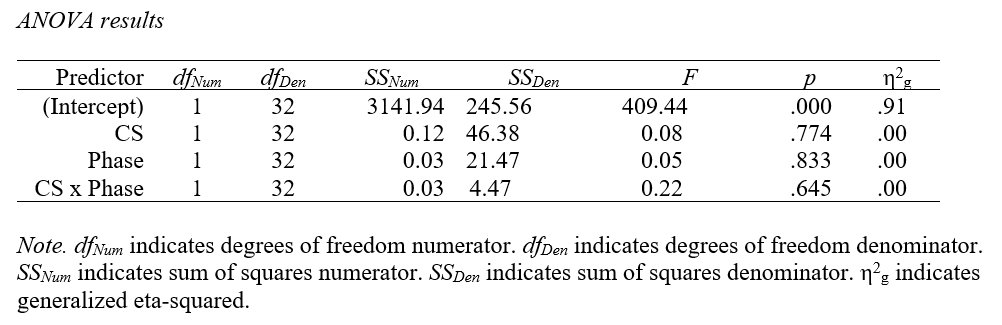


Table S6. Full table of ANOVA results using the effort data from Experiment 1. The 2x2 ANOVA consisted of CS (CS+ vs CS-) and Phase (Visual Acquisition Phase vs Visual Acquisition Phase)

##### 2.4.2 Effort Frequency Graph


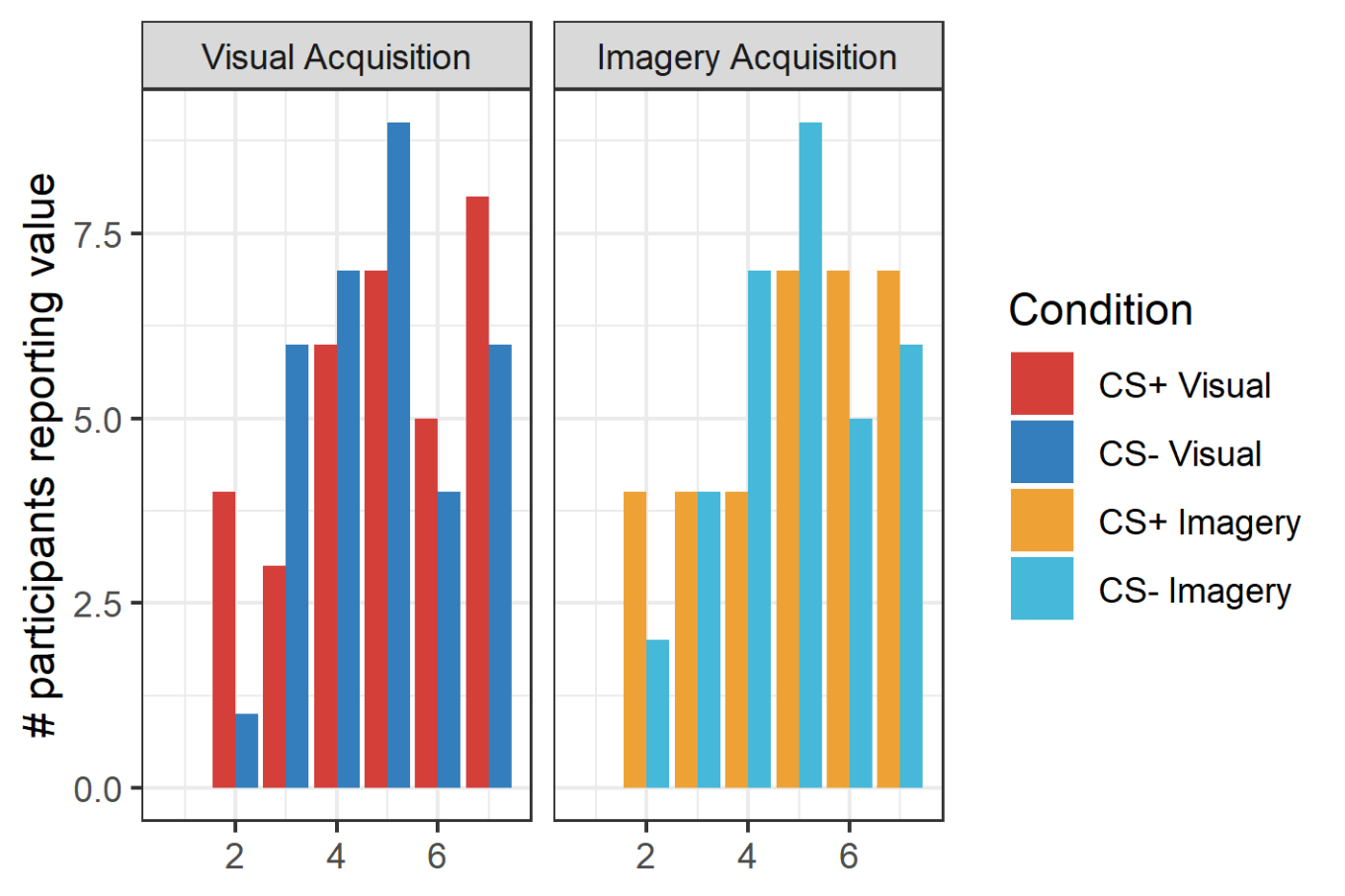


Fig. S8. Each bar represents the number of participants that reported the given value (1 (None Existent – 7 (Very Strong)) on the Likert-Style Questionnaire regarding effort used for each stimulus in Experiment 1. Red and dark blue bars represent imagining the CS+ and CS-, respectively, in the Visual Acquisition phase while orange and light blue bars represent imagining the CS+ and CS-, respectively, in the Imagery Acquisition phase. Note that the presence of a double wide bar indicates that that value was only chosen for one of the two conditions.

#### 2.5 SCR Data

##### 2.5.1 SCR Descriptive Data

|  | Visual Acquisition | | Imagery Acquisition | |
| --- | --- | --- | --- | --- |
|  | Mean | SD | Mean | SD |
| CS+ View | .43 | .44 | .32 | .23 |
| CS- View | .34 | .35 | .29 | .21 |
| CS+ Imagine | .35 | .26 | .50 | .42 |
| CS- Imagine | .28 | .22 | .33 | .24 |

Table S7. Descriptive statistics of SCR data of participants in Experiment 1 during both the Visual Acquisition phase and Imagery Acquisition phase

##### 2.5.2 SCR ANOVA Results


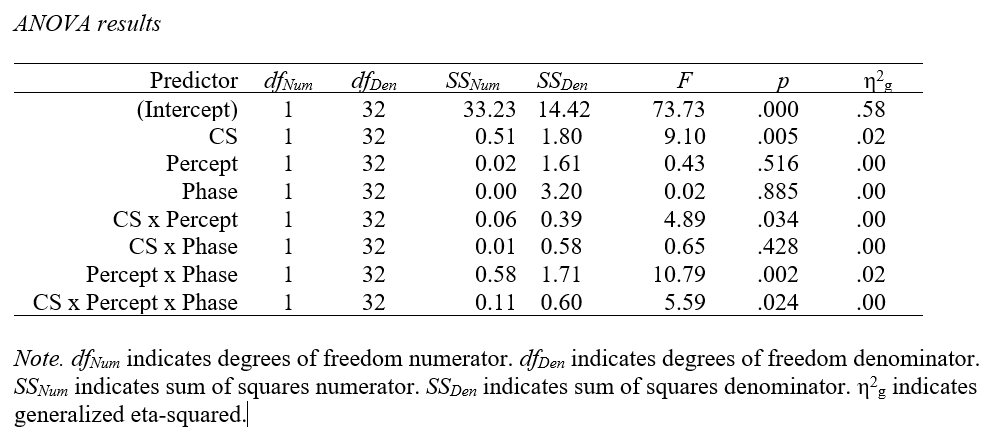


Table S8. Full table of ANOVA results using the SCR data from Experiment 1. The 2x2x2 ANOVA consisted of CS (CS+ vs CS-), Percept (viewed stimulus vs imagined stimulus), and Phase (Visual Acquisition Phase vs Visual Acquisition Phase)

##### 2.5.3 SCR Pairwise Comparison Results

In order to unpack the three-way interaction we conducted pairwise comparisons between the various conditions. Here we report those results that are not included in the main manuscript, as they are all non-significant.

Conditioning to the viewed CS+ in the Visual Acquisition phase was not significantly different from conditioning to the imagined CS+ in the Imagery Acquisition phase, *t*(32) = 1.09, *p* = .29. There was also no significant difference in SCR between the generalized percepts irrespective of the acquisition phase. The SCR to the imagined CS+ from the Visual Acquisition phase was not significantly different from that of the viewed CS+ from the Imagery Acquisition phase, *t*(32) = .917, *p* = .366.

Within the Visual Acquisition phase, the viewed CS- was not significantly different from the imagined CS-, *t*(32) = .36, *p* = .72. In the Imagery Acquisition phase the imagined CS- was not significantly different from the imagined CS-, *t*(32) = .56, *p* = .38.

The CS- conditions produced no significant difference between the imagined CS- from the Visual Acquisition phase and the viewed CS- from the Imagery Acquisition phase, *t*(32) = .31, *p* = .76. There was also no significant difference in the SCR between conditioning to a CS- view from Visual Acquisition phase versus generalizing to a CS- view from Imagery Acquisition phase, *t*(32) = 1.05, *p* = 0.30. Similarly, the conditioned response to a CS- imagine in the Imagery Acquisition phase was not significantly different from the generalization response to a CS- imagine from Visual Acquisition phase, *t*(32) = 1.14, *p* = 0.26. Likewise, there was no significant difference in SCR between the generalized percepts irrespective of the acquisition phase. The SCR to the CS- imagine from the Visual Acquisition phase was not significantly different from the SCR to the CS- view from Imagery Acquisition phase, *t*(32) = 0.10, *p* = 0.92. The SCR to the CS- view from the Visual Acquisition phase was also not significantly different from the SCR to the CS- imagine from Imagery Acquisition phase, *t*(32) = 0.31, *p* = 0.76.

##### 2.5.4 Bayesian Analysis SCR data

In the Visual Acquisition phase, the Bayesian Sensitivity Analysis of the SCR data for viewing the CS+ versus viewing the CS- resulted in: BF_10_(0.707) = 1.56, BF_10_(1) = 1.24, and BF_10_(1.41) = 0.94. The Sensitivity Analysis for imagining the CS+ versus imagining the CS- resulted in: BF_10_(0.707) = 1.94, BF_10_(1) = 1.55, and BF_10_(1.41) = 1.19.

In the Imagery Acquisition phase, the Bayesian Sensitivity Analysis assessing the participants’ SCR when imagining the CS+ versus imagining the CS- resulted in: BF_10_(0.707) = 11.30, BF_10_(1) = 9.70, and BF_10_(1.41) = 7.76. The Sensitivity Analysis assessing the participants’ fear when viewing the CS+ and viewing the CS- resulted in: BF_01_(0.707) = 3.71, BF_01_(1) = 5.07, and BF_01_(1.41) = 6.99.

##### 2.5.5 Temporal SCR Data - Visual Acquisition phase


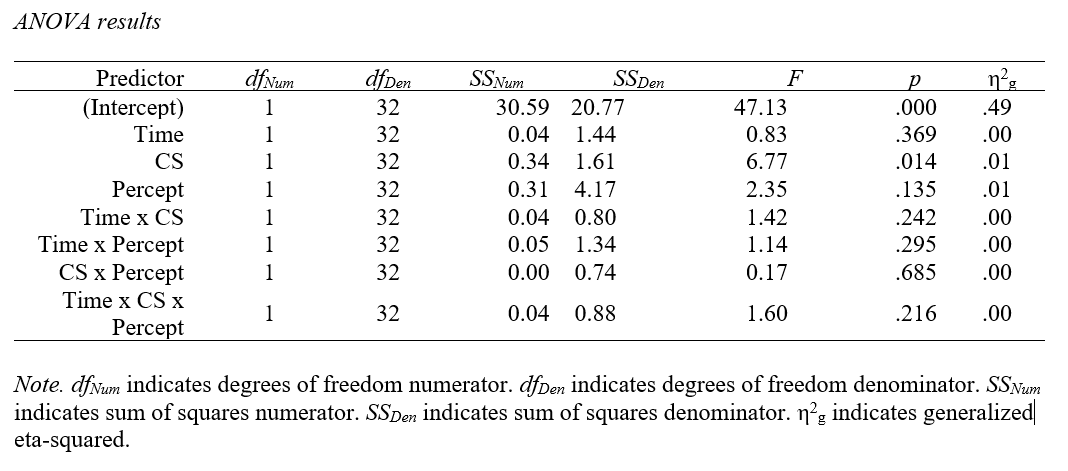


Table S9. Full table of ANOVA results using the SCR data from Experiment 1’s Visual Acquisition phase. The 2x2x2 ANOVA consisted of CS (CS+ vs CS-), Percept (viewed stimulus vs imagined stimulus), and Time (Early vs Late)

##### 2.5.6 Temporal SCR Data - Imagery Acquisition phase


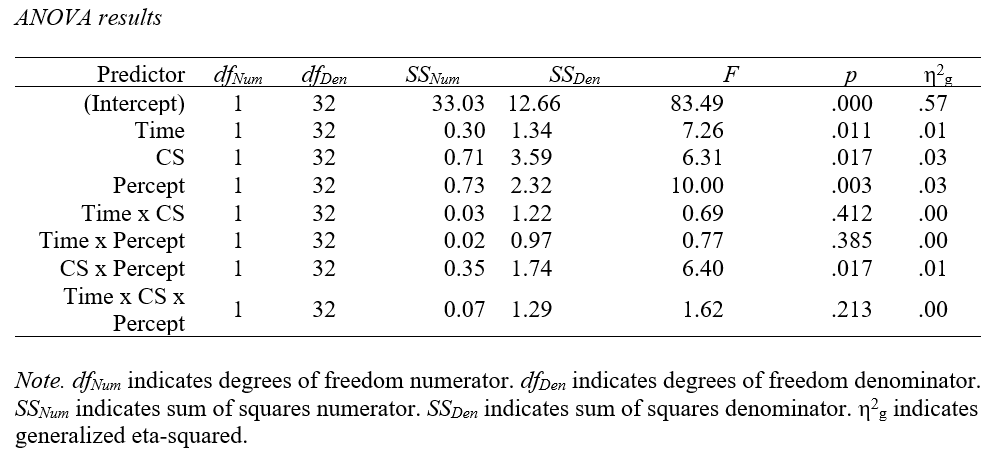


Table S10. Full table of ANOVA results using the SCR data from Experiment 1’s Imagery Acquisition phase. The 2x2x2 ANOVA consisted of CS (CS+ vs CS-), Percept (viewed stimulus vs imagined stimulus), and Time (Early vs Late)

####

### 3 Experiment 2

#### 3.1 Subjective Questionnaires

|  | Mean | SD |
| --- | --- | --- |
| VVIQ | 65.46 | 8.86 |
| State Anxiety Inventory | 40.54 | 6.18 |
| Trait Anxiety Inventory | 42.27 | 11.40 |
| Attentional Control Scale | 53.48 | 9.93 |

Table S11. Descriptive statistics of questionnaires completed by participants in Experiment 2

#### 3.2 Self-Reported Fear

##### 3.2.1 Likert-style Descriptive Data

|  | Mean | SD |
| --- | --- | --- |
| Vividness: CS+ imagine | 5.81 | 1.04 |
| Vividness: CS- imagine | 5.41 | 1.37 |
| Effort: CS+ imagine | 6.07 | 1.24 |
| Effort: CS- imagine | 5.93 | 1.27 |
| Fear: CS+ view | 4.67 | 1.98 |
| Fear: CS- view | 1.93 | 1.38 |
| Fear: CS+ imagine | 4.22 | 0.50 |
| Fear: CS- imagine | 2.19 | 0.83 |

Table S12. Descriptive statistics of self-reported, likert-style questionnaire by participants in Experiment 2

##### 3.2.2 Self-Reported Fear ANOVA Results


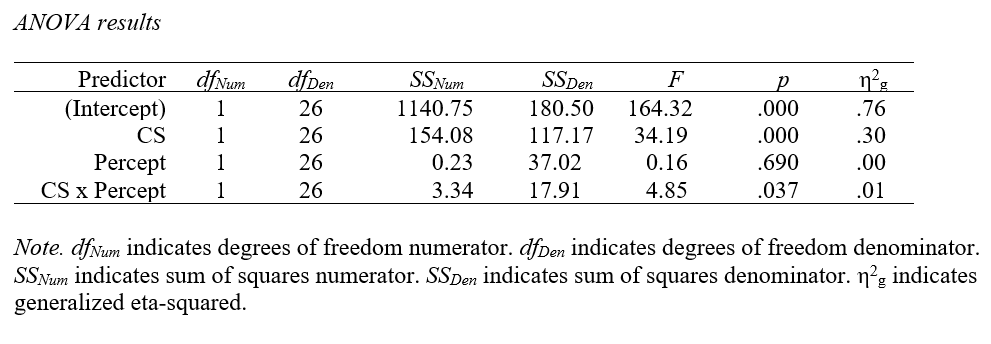


Table S13. Full table of ANOVA results using the self-reported fear data from Experiment 2. The 2x2 ANOVA consisted of CS (CS+ vs CS-) and Imagery (viewed stimulus vs imagined stimulus).

##### 3.2.3 Self-Reported Fear Data for All Participants


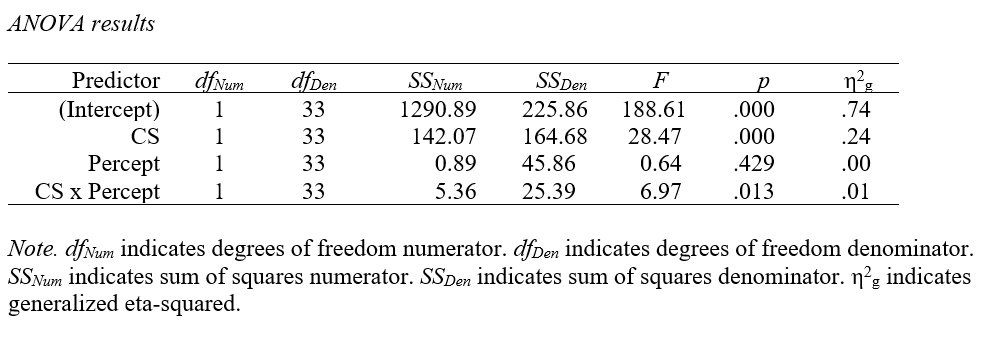


Table S14. Full table of ANOVA results using the self-reported fear data from all participants in Experiment 2. This table includes participants who’s SCRs were deemed as too noisy or non-responders. The 2x2 ANOVA consisted of CS (CS+ vs CS-) and Imagery (viewed stimulus vs imagined stimulus).

#####

##### 3.2.4 Self-Reported Fear Pairwise Comparison Results

In order to unpack the interaction we conducted pairwise comparisons between the various conditions. Here we report those results that are not included in the main manuscript, as they are all non-significant.

In order to verify that participants did not have an increased self-reported fear of either CS-, a t-test was conducted to compare the CS-s. This test was not significant, indicating that there was no difference in base fear towards either patch, *t*(26) = 1.49, *p* = .15. Participants also had no significant difference in self-reported fear for the CS+s, *t*(26) = 1.25, *p* = .22

##### 3.2.5 Bayesian Analysis Self-Reported Fear data

The Sensitivity Analysis assessing the participants’ fear when viewing the CS+ and viewing the CS- resulted in: BF_10_(0.707) = 6806.17, BF_10_(1) = 7327.34, and BF_10_(1.41) = 7101.99. Likewise, the Sensitivity Analysis assessing the participants’ generalized fear when imagining the CS+ and imagining the CS- resulted in: BF_10_(0.707) = 568.90, BF_10_(1) = 573.74, and BF_10_(1.41) = 523.07.

##### 3.2.6 Vividness and Effort Frequency Graph


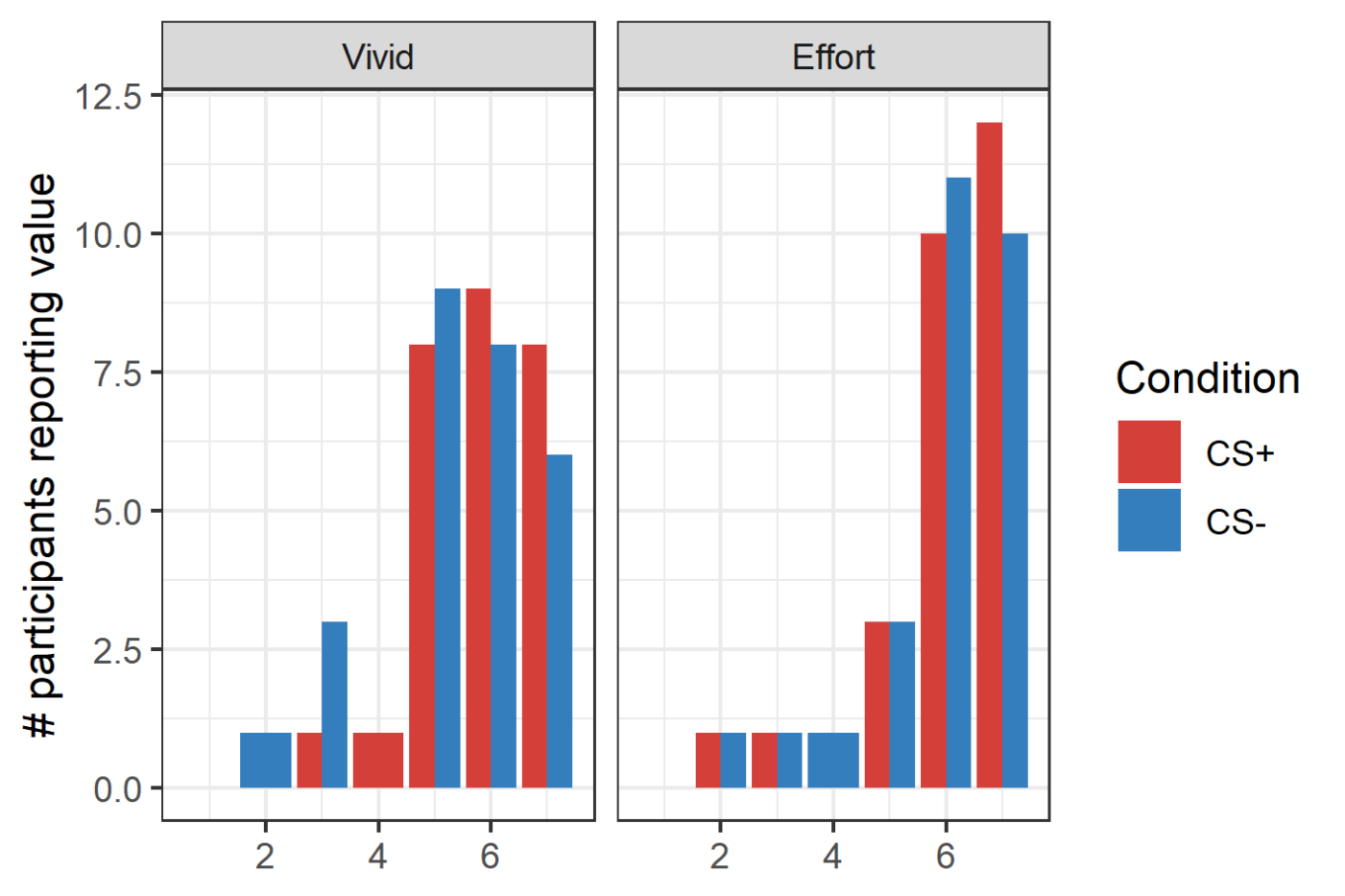


Fig. S9. Each bar represents the number of participants that reported the given value (1 (None Existent – 7 (Very Strong)) on the Likert-Style Questionnaire regarding vividness (left) and effort (right) used for each stimulus in Experiment 2. Red and dark blue bars represent imagining the CS+ and CS-, respectively. Note that the presence of a double wide bar indicates that that value was only chosen for one of the two conditions.

#### 3.3 SCR Data

##### 3.3.1 SCR Descriptive Data

|  | Mean | SD |
| --- | --- | --- |
| CS+ View | .33 | .32 |
| CS- View | .24 | .21 |
| CS+ Imagine | .38 | .25 |
| CS- Imagine | .35 | .28 |

Table S15. Descriptive statistics of SCR data of participants in Experiment 2

##### 3.3.2 SCR ANOVA Results


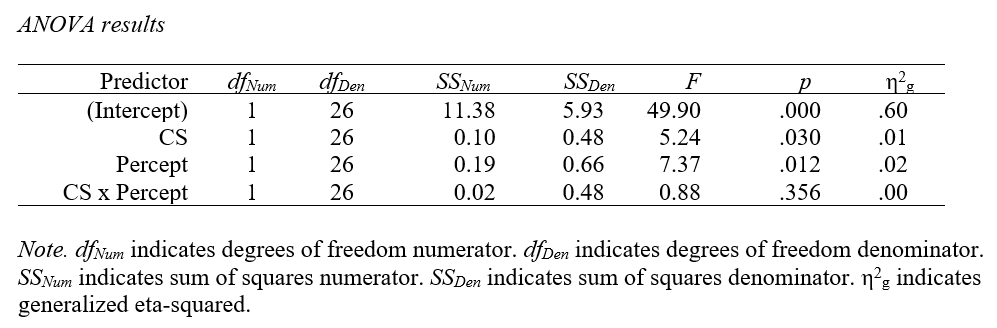


Table S16. Full table of ANOVA results using the SCR data from Experiment 2. The 2x2 ANOVA consisted of CS (CS+ vs CS-) and Imagery (viewed stimulus vs imagined stimulus).

##### 3.3.3 Bayesian Analysis SCR data

The Sensitivity Analysis assessing the participants’ fear when viewing the CS+ and viewing the CS- resulted in: BF_10_(0.707) = 1.41, BF_10_(1) = 1.13, and BF_10_(1.41) = 0.087. The Sensitivity Analysis Assessing the participants’ fear when imagining the CS+ and imagining the CS- resulted in: BF_10_(0.707) = 0.33, BF_10_(1) = 0.25, and BF_10_(1.41) = 0.18. The Sensitivity Analysis Assessing the participants’ fear regardless of perception resulted in: BF_10_(0.707) = 1.86, BF_10_(1) = 1.51, and BF_10_(1.41) = 1.17.

#####

##### 3.3.4 Temporal SCR ANOVA


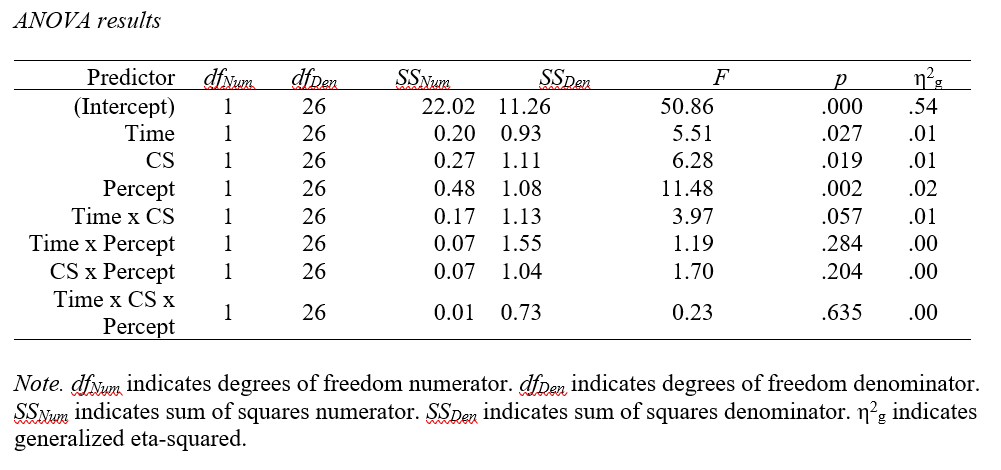


Table S17. Full table of ANOVA results using the SCR data from Experiment 2. The 2x2x2 ANOVA consisted of CS (CS+ vs CS-), Percept (viewed stimulus vs imagined stimulus), and Time (Early vs Late)

### 4 Experiment 3

#### 4.1 Self-Report Questionnaire Data

|  | Mean | SD |
| --- | --- | --- |
| VVIQ | 65.50 | 7.73 |
| State Anxiety Inventory | 39.19 | 7.50 |
| Trait Anxiety Inventory | 39.31 | 12.63 |
| Attentional Control Scale | 48.89 | 9.49 |

Table S18. Descriptive statistics of questionnaires completed by participants in Experiment 3

####

#### 4.2 Self-Reported Fear

##### 4.2.1 Likert-style Data

|  | Mean | SD |
| --- | --- | --- |
| Vividness: CS+ imagine | 5.38 | 1.42 |
| Vividness: CS- imagine | 5.35 | 1.60 |
| Effort: CS+ imagine | 5.08 | 1.81 |
| Effort: CS- imagine | 5.00 | 1.81 |
| Fear: CS+ view | 3.31 | 1.64 |
| Fear: CS- view | 2.23 | 1.63 |
| Fear: CS+ imagine | 4.62 | 1.72 |
| Fear: CS- imagine | 2.73 | 1.80 |

Table S19. Descriptive statistics of self-reported, likert-style questionnaire by participants in Experiment 3

##### 4.2.2 Self-Reported Fear ANOVA Results


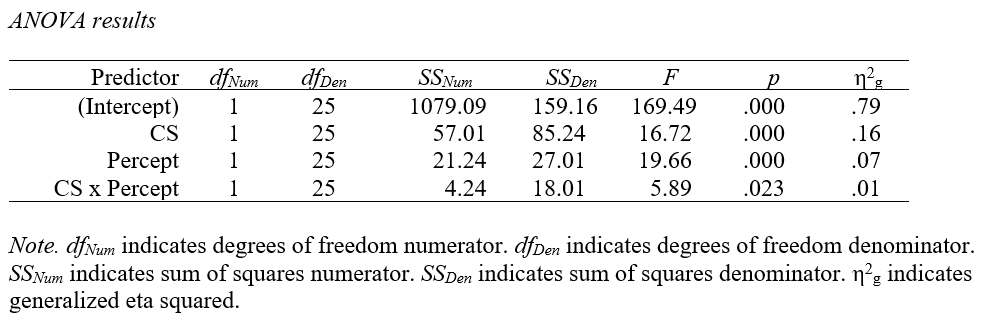


Table S20. Full table of ANOVA results using the self-reported data from Experiment 3. The 2x2 ANOVA consisted of CS (CS+ vs CS-) and Imagery (viewed stimulus vs imagined stimulus).

##### 4.2.3 Self-Reported Fear ANOVA Results for All Participants


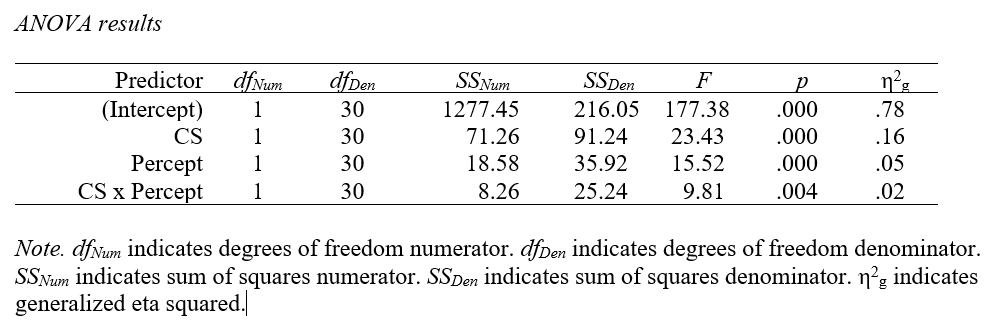


Table S21. Full table of ANOVA results using the self-reported data from all participants in Experiment 3. This table includes participants who’s SCRs were deemed as too noisy or non-responders. The 2x2 ANOVA consisted of CS (CS+ vs CS-) and Imagery (viewed stimulus vs imagined stimulus).

##### 4.2.4 Bayesian Analysis Self-Reported Fear data

The Sensitivity Analysis assessing the participants’ fear when imagining the CS+ and imagining the CS- resulted in: BF_10_(0.707) = 63.18, BF_10_(1) = 59.77, and BF_10_(1.41) = 51.64. The Sensitivity AnalysisAssessing the participants’ fear when viewing the CS+ and viewing the CS- resulted in: BF_10_(0.707) = 20.51, BF_10_(1) = 18.60, and BF_10_(1.41) = 15.55.

##### 4.2.5 Vividness and Effort Frequency Graph


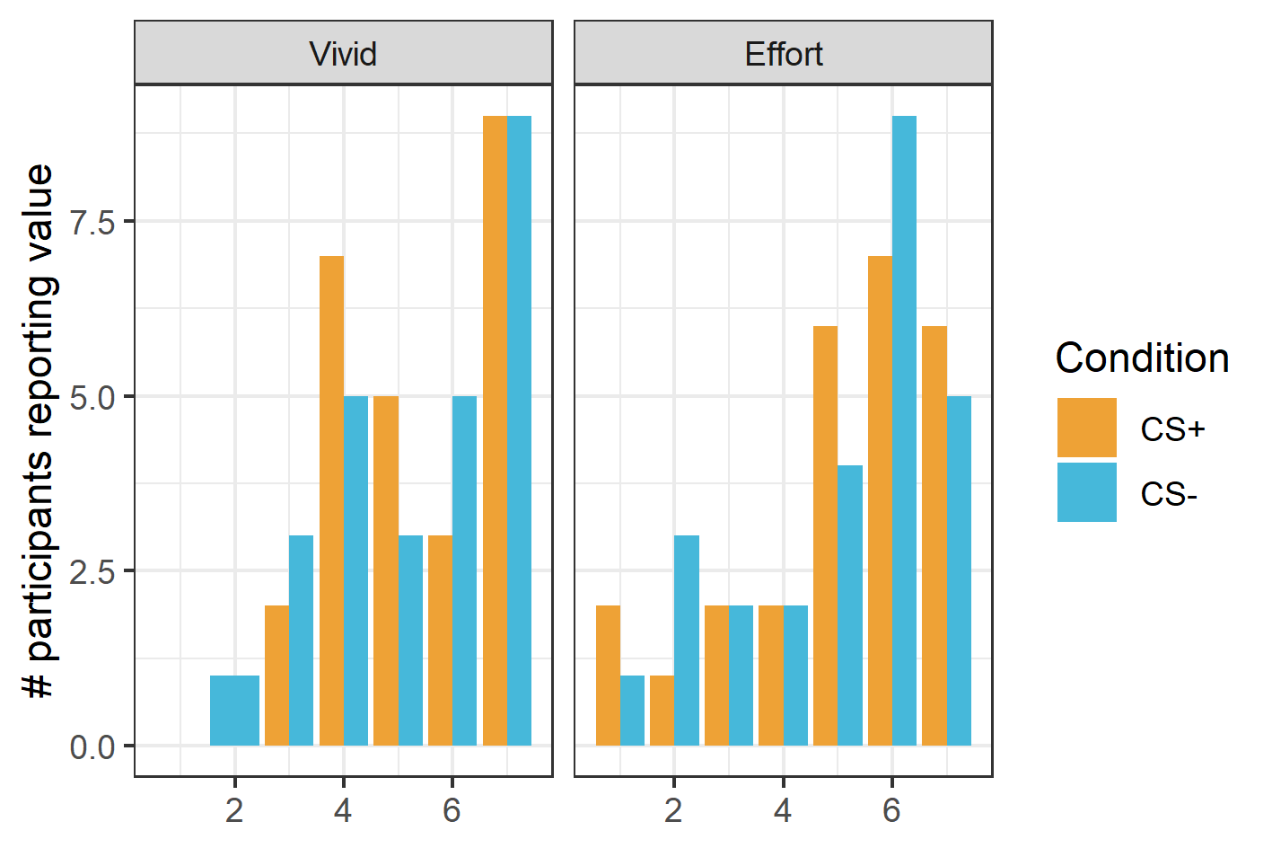


Fig. S10. Each bar represents the number of participants that reported the given value (1 (None Existent – 7 (Very Strong)) on the Likert-Style Questionnaire regarding vividness (left) and effort (right) used for each stimulus in Experiment 3. Orange and light blue bars represent imagining the CS+ and CS-, respectively.

#### 4.3 SCR

##### 4.3.1 SCR Data

|  | Mean | SD |
| --- | --- | --- |
| CS+ View | .37 | .19 |
| CS- View | .22 | .18 |
| CS+ Imagine | .71 | .39 |
| CS- Imagine | .45 | .31 |

Table S22. Descriptive statistics of SCR data of participants in Experiment 3

##### 4.3.2 SCR ANOVA Results


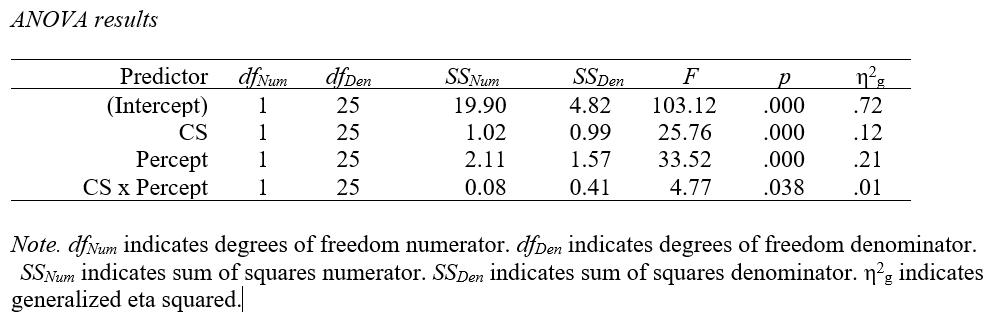


Table S23. Full table of ANOVA results using the SCR data from Experiment 3. The 2x2 ANOVA consisted of CS (CS+ vs CS-) and Imagery (viewed stimulus vs imagined stimulus).

##### 4.3.3 Bayesian Analysis SCR data

The Sensitivity Analysis assessing the participants’ differential SCR when imagining the CS+ and imagining the CS- resulted in: BF_10_(0.707) = 409.45, BF_10_(1) = 412.09, and BF_10_(1.41) = 375.18. Regarding the generalized SCR, viewing the CS+ versus viewing the CS- resulted in the following Sensitivity Analysis: BF_10_(0.707) = 30.32, BF_10_(1) = 27.91, and BF_10_(1.41) = 23.60.

#####

##### 4.3.4 Temporal SCR Data


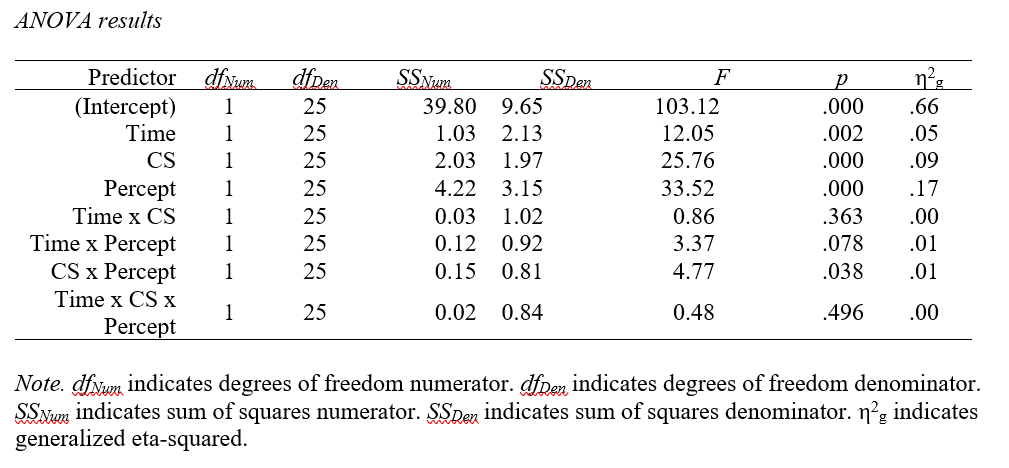


Table S24. Full table of ANOVA results using the SCR data from Experiment 3. The 2x2x2 ANOVA consisted of CS (CS+ vs CS-), Percept (viewed stimulus vs imagined stimulus), and Time (Early vs Late)
